# Signal Transduction and Pathogenic Modifications at the Melanocortin-4 Receptor: A Structural Perspective

**DOI:** 10.3389/fendo.2019.00515

**Published:** 2019-07-31

**Authors:** Nicolas Heyder, Gunnar Kleinau, Michal Szczepek, Dennis Kwiatkowski, David Speck, Lucia Soletto, José Miguel Cerdá-Reverter, Heiko Krude, Peter Kühnen, Heike Biebermann, Patrick Scheerer

**Affiliations:** ^1^Group Protein X-ray Crystallography and Signal Transduction, Institute of Medical Physics and Biophysics, Berlin Institute of Health, Charité – Universitätsmedizin Berlin, Corporate Member of Freie Universität Berlin, Humboldt-Universität zu Berlin, Berlin, Germany; ^2^Departamento de Fisiología de Peces y Biotecnología, Consejo Superior de Investigaciones Científicas, Instituto de Acuicultura Torre de la Sal, Ribera de Cabanes, Spain; ^3^Institute of Experimental Pediatric Endocrinology, Berlin Institute of Health, Charité – Universitätsmedizin Berlin, Corporate Member of Freie Universität Berlin, Humboldt-Universität zu Berlin, Berlin, Germany

**Keywords:** G-protein coupled receptor, melanocortin receptors, MC4R, MSH, AgRP, ACTH, pathogenic mutations

## Abstract

The melanocortin-4 receptor (MC4R) can be endogenously activated by binding of melanocyte-stimulating hormones (MSH), which mediates anorexigenic effects. In contrast, the agouti-related peptide (AgRP) acts as an endogenous inverse agonist and suppresses ligand-independent basal signaling activity (orexigenic effects). Binding of ligands to MC4R leads to the activation of different G-protein subtypes or arrestin and concomitant signaling pathways. This receptor is a key protein in the hypothalamic regulation of food intake and energy expenditure and naturally-occurring inactivating *MC4R* variants are the most frequent cause of monogenic obesity. In general, obesity is a growing problem on a global scale and is of social, medical, and economic relevance. A significant goal is to develop optimized pharmacological tools targeting MC4R without adverse effects. To date, this has not been achieved because of *inter alia* non-selective ligands across the five functionally different MCR subtypes (MC1-5R). This motivates further investigation of (i) the three-dimensional MC4R structure, (ii) binding mechanisms of various ligands, and (iii) the molecular transfer process of signal transduction, with the aim of understanding how structural features are linked with functional-physiological aspects. Unfortunately, experimentally elucidated structural information is not yet available for the MC receptors, a group of class A G-protein coupled receptors (GPCRs). We, therefore, generated MC4R homology models and complexes with interacting partners to describe approximate structural properties associated with signaling mechanisms. In addition, molecular insights from pathogenic mutations were incorporated to discriminate more precisely their individual malfunction of the signal transfer mechanism.

## Introduction

The melanocyte-stimulating hormones (α-, β-, γ-MSH) and the adrenocorticotropic hormone (ACTH) are agonistic peptidic ligands that bind to a group of five class A GPCRs (1), namely the melanocortin-receptors 1–5 (MC1-5R). In contrast, the endogenous melanocortin receptor antagonist AgRP (2–4) inhibits basal constitutive signaling (5) as a potential inverse agonist and simultaneously prevents MSH binding. These ligands and receptors are involved in regulating various physiological functions, such as skin pigmentation, energy homeostasis, erythrocyte differentiation, thermal homeostasis, appetite, and adrenal, or sexual function (6–9). MCRs are expressed in many tissues and mutations in MCRs cause pathogenic conditions such as analgesia, obesity, hypocortisolism, and inflammation (4, 10). The MC2R, in contrast to other MCRs, is specific because it is activated only by ACTH and requires an interplay with the melanocortin receptor accessory protein (MRAP) to attain functional expression (11). MC2R activation is associated with stress responses by promoting the synthesis and secretion of adrenal glucocorticoids along the hypothalamic-pituitary-adrenal axis (12).

The MC4R has a fundamental role in regulating food intake and energy expenditure (13, 14). It is expressed primarily in the hypothalamic paraventricular nucleus, spinal cord, sympathetic preganglionic neurons, and brainstem (15, 16). MC4R mainly couples to the G-protein Gs (17) as a cAMP-dependent pathway activator, but can also activate other G-protein subtypes such as Gq or Gi (18, 19). Agonist-mediated and basal (constitutive) MC4R signaling activity was proposed to differentially impact various N-type voltage-gated calcium channels (CaV) through Gs and Gi/o pathways. In addition, chronic incubation with AgRP occludes CaV inhibition (20). Moreover, regulation of neuronal firing activity from the paraventricular nucleus of the hypothalamus (PVN) by α-MSH and AgRP can be mediated by the inwardly rectifying potassium channel, Kir7.1 (21).

Agonistic action at MC4R induces an anorexigenic effect (appetite reducing) (22) in contrast to the antagonistic ligand AgRP with orexigenic effects (19). To date, inactivating *MC4R* mutations (23) are the most frequent monogenic cause of obesity (24). Currently, there are estimated to be more than 650 million adults with obesity worldwide (25). As obesity is related to different comorbidities such as diabetes mellitus type 2 or cardiovascular disease (26), there is considerable medical, pharmacological, as well as economic interest concerning this receptor (27). The design of highly selective and potent MC4R ligands (28, 29) should be a tool to counteract specifically against obesity (9, 30), which also needs a comprehensive understanding of this receptor under structural-functional perspectives.

In recent years, a large amount of information on the MC4R was gained including the identification of the interacting and signaling regulating melanocortin-2 receptor accessory protein (MRAP2) (31, 32), a newly discovered endogenously acting ligand lipocalin-2 (33), highly efficient synthetic, or peptidic ligands such as setmelanotide [termed RM-493, previously BIM-22493, IRC-022493] (23, 34–36), or bivalent ligands [e.g., (37–41)]. In addition, this receptor constitutes protein oligomers that are of functional importance (42–44) and few pathogenic MC4R variants have been reported to be dominant-negative on wild-type receptor function (42, 45). Finally, as already shown for other MCRs such as the MC1R and MC5R (46), heteromerization has been demonstrated for MC4R, which is consequently a further modulatory factor in the MC4R interaction network (47, 48).

Interestingly, several pathogenic MC4R mutations have been observed to cause biased signaling through the MC4R by inducing a preference for a specific signaling pathway. Moreover, AgRP can simultaneously induce or block different signaling pathways and newly developed biased MC4R ligands were applied as an anti-obesity treatment (19, 49, 50).

These insights indicate an elaborate and complex regulatory mechanism of the MC4R on a molecular and cellular level, with a multitude of interacting proteins, and (patho-) physiological relationships (51). Unfortunately, structural information on the MCR subtypes is not yet available, hampering our understanding of this functional information. Therefore, in the present study, we generated and used structural models of ligand/MC4R complexes as well as a ligand/MC4R/G-protein ternary complex to evaluate general and local features of signaling-related processes under the perspective of structure-function relationships. Finally, this also aids in improving our understanding of pathogenic mutations in receptor activation at the molecular level.

## Methods

### Modeling of the Human MC4R in an Inactive State Conformation

**Figure 1:**
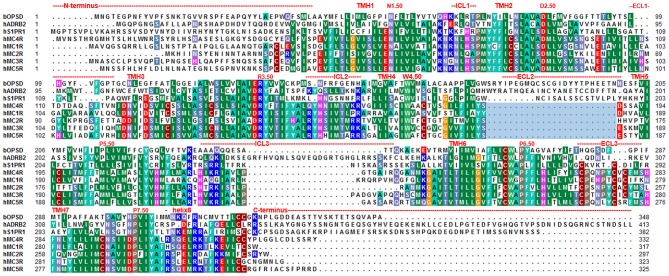
Amino acid sequence alignment between MCR subtypes and potential structural templates for MC4R homology modeling in different activity states. The sequence alignment shows overlapping or diverse properties between the five MCR subtypes, the sphingosine 1-phosphate receptor 1 [S1PR1, template for inactive state MC4R homology models, PDB entry 3V2Y (53)], the prototypical class A rhodopsin, and the β-adrenergic receptor [ADRB2, template for active state MC4R homology model, PDB entry 3SN6, (54)]. Highly conserved positions according to the *Ballesteros and Weinstein* numbering scheme (55) are indicated by respective numbers. The very short ECL2 of the MCR group compared to other GPCRs is highlighted by a light blue rectangle. Predicted structural dimensions of each receptor segment are indicated above the sequences. The alignment was visualized using the software BioEdit (56). Specific background colors indicating conservation (Blossum62 matrix) among different receptors and reflecting chemical properties of the amino acid side-chains: black, proline; blue, positively charged; cyan/green, aromatic and hydrophobic; green, hydrophobic; red, negatively charged; gray, hydrophilic; dark red, cysteines; and magenta, histidine.

The computational modeling procedure of the human MC4R (hMC4R) in an inactive state conformation was recently described in detail by our group (52). Briefly, the lysophospholipid sphingosine 1-phosphate receptor structure [S1PR1, PDB entry 3V2Y) (53)], which has a high sequence similarity (~50%) to hMC4R in the transmembrane region, was used (Figure 1). Additionally, this template shows specific overlapping features with the MC4R, e.g., S1PR1 is characterized by a leucine in transmembrane helix (TMH) 5 position 5.50 [according to the Ballesteros & Weinstein numbering system (55)] and consequently has a regular α-helical conformation, which is expected for the MC4R due to the presence of methionine at the corresponding position (Figure 1). In most other class A GPCRs, TMH5 is kinked due to the location of proline at position 5.50.

The disulfide bridge between extracellular loop (ECL) 2 and transmembrane helix (TMH) 3 is missing in S1PR1 and is also assumed for the MC4R (absence of cysteine at the respective positions). A disulfide bridge is present in ECL3 and also proposed for the MC4R (57).

The N-terminus of S1PR1 comprises 40 amino acids compared to 39 amino acids in hMC4R. Template preparation included loop length adjustments. Missing residues between the N-terminal helix and TMH1 (between A39–L47) were added manually. Amino acids in the S1PR1 template were substituted with residues of the hMC4R according to the sequence alignment between S1PR1 and hMC4R (Figure 1), followed by energy minimization of side chains. This preliminary model was refined by molecular dynamic simulations of side chains and loops, succeeded by energy minimization until converging at a termination gradient of 0.05 kcal/mol^*^Å. The AMBER (58) F99 force field was used for energy minimization and dynamic simulations included in *Sybyl X2.0* (Certara, NJ, US).

### Ligand Models

While many MCR ligands, synthetic or endogenous, are already known (59, 60), we here focused only on a small subset of endogenous native ligands such as alpha-MSH or AgRP to link structural models with the different aspects of MC4R signaling and regulation. We recently described a modeling and docking procedure for the hMC4R in a complex with the agonistic peptide ligands α-MSH and setmelanotide (50). The MC4R/α-MSH complex model was used to compare the suggested binding mode with the here inferred binding mode between hMC4R and AgRP, zfMC4R with MSH, but also to visualize putative MC4R oligomer arrangements and to map amino acid positions of pathogenic mutants. For AgRP, a previously determined structure of a functionally active protein fragment is available [PDB entry 1HYK (61)] with amino acids between the positions 87–132. Of note, further information on peptidic ligand structures has been published previously (62).

### Ligand/Receptor Complex-Assembling

For ligand docking of AgRP into the inactive MC4R model (see section Ligand models) the known structural AgRP fragment (see section Ligand models) was placed manually in the extracellular solvent phase at approximately 5 Å separation from the hMC4R surface in close spatial proximity above the extracellular loops. A known interaction between the amino acids of the ligand and the receptor involved in binding (16, 63–66) was used as a distance constraint. In particular, the specific AgRP motif _111_RFFN_114_ is essential for interaction with the orthosteric site of the MC4R and the MC3R (67, 68). In accordance to others [reviewed in Ericson et al. (9)], we thereby assumed that the positively charged side chain of R111 (AgRP) interacts with the negatively charged side chains of D122 and D126 (MC4R). Molecular dynamic simulations (300 K, 3 ns) were initiated with a distant constraint of 2 Å between the side chains of AgRP R111 and hMC4R D126. All backbone atoms of the receptor helices and the ligand were constrained. The resulting model was energetically minimized, followed by a second dynamic simulation (2 ns) without any distance constraints on the ligand backbone. The resulting complex model was energetically minimized without any constraint.

### A Receptor/Ligand/G-Protein Ternary Complex Model

Our recently described hMC4R/α-MSH complex model (50), based on the determined active state β-adrenergic receptor (ADRB2) structure as a complex with heterotrimeric Gs [PDB entry 3SN6 (54)], was superimposed with the initial receptor/Gs complex template. The heterotrimeric G-protein from the template was substituted into the MC4R/ligand complex model. Dynamic simulations (300 K, 2 ns) of the side chains and loop structures were used to optimize interactions and intramolecular distances in the complex, whereby the backbone atoms of the receptor helices were constrained. The resulting model was energetically minimized without any structural constraint.

### Homology Modeling of Complexes Between Zebrafish MC2R or zfMC4R and Agonistic Peptide Ligands

Our recently published hMC4R homology as a complex with α-MSH (50) was used as a template for modeling the zebrafish (zf) MC4R as a complex with α-MSH, but also for the zfMC2R bound with agonistic ACTH. The purpose was to evaluate the possibility of similar ligand binding modes at these receptors on a structural level and to suppose a potential mechanism of action for ACTH at zfMC2R. This can be reasoned by the identification of two MRAP paralogue genes in zebrafish, zfMRAP2a, and zfMRAP2b (69), with zfMRAP2a found to increase the zfMC4R response to ACTH, most likely by heteromerization [Josep et al. (70)]. Consequently, zfMC4R becomes an ACTH receptor in the presence of MRAP2a and similar results have been reported recently in chickens (60) and also for the human MC4R (71) in interaction with MRAP2. Therefore, our complex models should help to generate functional mechanistic hypotheses concerning increased ACTH sensitivity at zfMC4R mediated by interaction with MRAP2a, although we cannot offer a concrete heteromeric MC4R/MRAP2 complex model because of missing structural information on MRAP (no valuable template or elucidated structure is available).

The overall sequence identity between zfMC4R and hMC4R (Supplementary Figure 1) is 68%, whereas the sequence similarity between zfMC4R and zfMC2R is ~60% (the Blossum62 matrix was applied). The hMC4R model was used as a template and the zfMC4R amino acids were substituted into this template, followed by energy minimization of side chains. The modeling and docking procedure of α-MSH to hMC4R was previously described by our group (50), with the identical procedure used to dock α-MSH into the zfMC4R.

Structural modifications to adapt the hMC4R model toward a three-dimensional zfMC2R structure (e.g., deletion of amino acids in the N-terminus (Ntt) and concomitant amino acid sequence substitutions) were performed using the software *Sybyl X2.0* (Certara, NJ, US). Moreover, ACTH has an N-terminal sequence that is identical with α-MSH, but the consecutive residues of the extended ACTH are reported to act antagonistically at MC2R as a single peptide (51). Because the structure of ACTH has not yet been determined, the MSH model was used as a template (positions 1–12) and functionally relevant amino acids were added manually from positions 13–24. The essential amino acids covering the binding site of MSH are conserved between hMC4R, zfMC4R, and zfMC2R (Supplementary Figure 1). Therefore, the hormone model was placed into the zfMC2R model with the “MSH-moiety” as supposed from the zfMC4R/MSH complex. The conformation and binding mode of ACTH in zfMC2R were adapted during 1 ns molecular dynamics of the complex [Amber force field (58)] with fixed backbone atoms of receptor helices and of ligand amino acids 1–12. The entire model-complex was energetically minimized without constraint.

### Dimeric and Oligomeric MC4R Complexes

The MC4R has the capacity to constitute homodimers (42–44). Functional data based on particular MC4R constructs suggest that specific parts of TMH3, intracellular loop 2 (ICL2), and TMH4 (TMH3- ICL2-TMH4) are involved in the interface between the receptor protomers (72). MC4R homodimerization is of functional importance with regard to signaling capacity (72). Based on previously determined crystal structures of class A GPCR dimers [reviewed in Audet and Bouvier (73)] as well as considerable biophysical studies, several oligomeric GPCR protomer arrangements were previously suggested (details described below). Consequently, dimeric GPCR constellations with different protomer interfaces can be predicted for GPCR dimers in the absence of structural information and GPCR crystal structures are useful to serve as templates for dimeric GPCR homology models [reviewed in Audet and Bouvier (73)].

Two putative oligomeric MC4R models were generated by superimposition of protomers (MC4R/α-MSH complex) with structural templates representing the two most commonly observed dimer arrangements [reviewed in Katritch et al. (74) and Baltoumas et al. (75)]. First, an MC4R homodimer with an interface between parts of TMH4, ICL2, and TMH5, based on the dimeric crystal structure of C-X-C chemokine receptor type 4 (CXCR4), was constructed. Second, an MC4R dimer model based on the human κ-opioid receptor (KOR) crystal structure was designed. These two different MC4R-MC4R dimeric complex models were sterically and energetically optimized using dynamic simulations (1 ns) of the side chains and loop structures, whereby the backbone atoms of the receptor helices were constrained. The resulting model was energetically minimized without structural constraint.

## Results and Discussion

### The Putative Three-Dimensional MC4R Protein Structure

**Figure 2:**
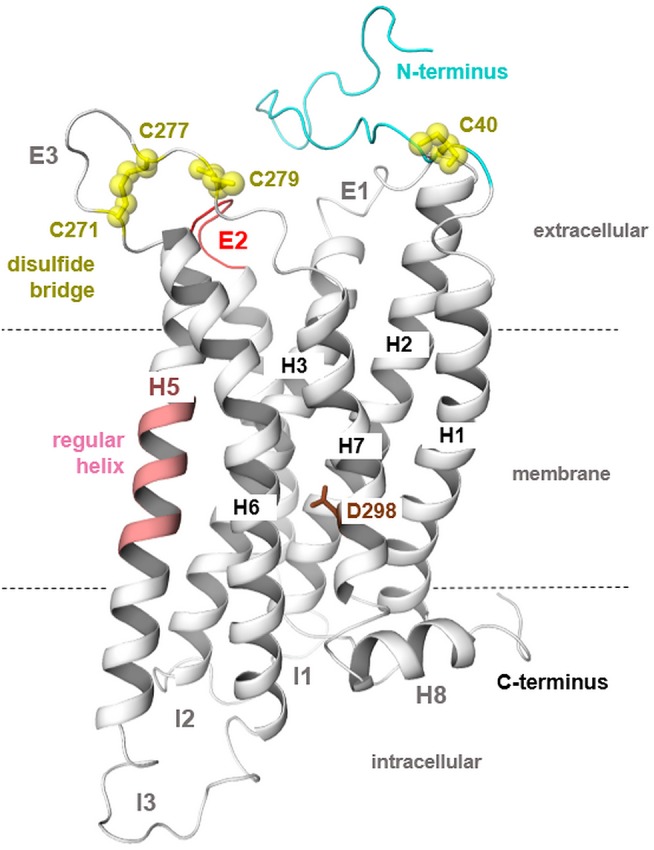
MC4R homology model in a putative inactive state. The three-dimensional hMC4R model (protein backbone as a cartoon) represents predicted general and specific structural features. First, the TMH5 in most class A GPCRs is characterized by a bulge and kink caused by occurrence of a highly conserved proline (P_5.50_); however, in the MCRs, there is a methionine instead of a proline at this position (Figure 1). This suggests a regular α-helical conformation (light magenta) for TMH5 as also observed in the structural template for the inactive state modeling [S1PR1, PDB entry 3V2Y (53)]. Second, the ECL2 (red) of all MCRs is extremely short in length compared to other GPCRs. Moreover, the highly conserved disulfide bridge in GPCRs between two cysteines located in ECL2 and the TMH3 is absent because of missing cysteines at respective positions. In the ECL3, two cysteines constitute a disulfide bridge (57). The N-termini of MCRs do not demonstrate overlapping in the sequence comparison (Figure 1); however, there has been discussion on the involvement of a specific N-terminal region in signaling regulation in the MC4R (77, 78). Finally, the conserved NPxxY motif in the TMH7 is a DPxxY motif with different chemical properties for this microdomain in the MC4R.

**Figure 3:**
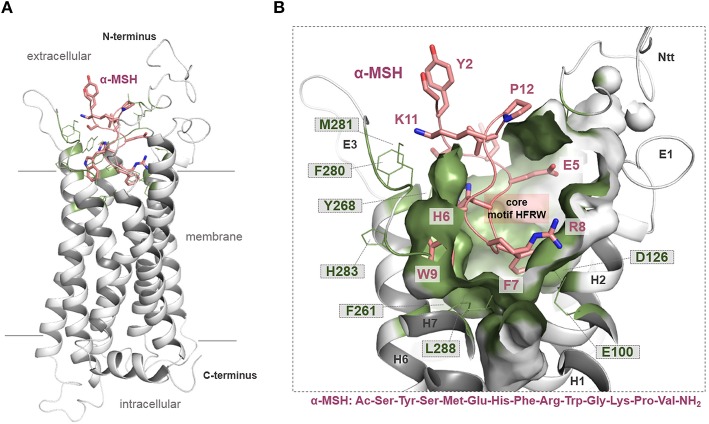
MC4R in interaction with native agonist α-MSH. **(A)** The human hMC4R/α-MSH complex model reveals that the peptide agonist binds into a cleft between the extracellular loops and transmembrane helices. **(B)** More than twenty hMC4R amino acids (examples labeled) constituting the putative ligand binding pocket (inner surface, clipped, green). In α-MSH, the central recognition motif _6_HFRW_9_ is involved in mediating ligand induced effects.

**Figure 4:**
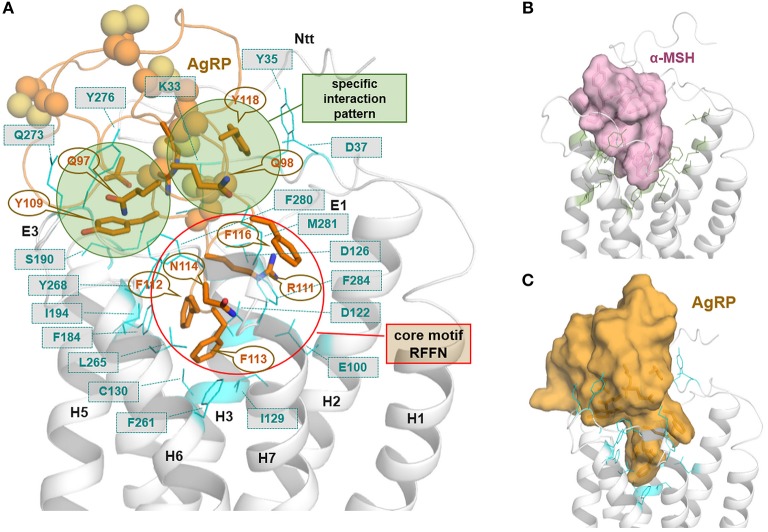
MC4R in interaction with the endogenous inverse agonist AgRP. The available solved structure (determined by NMR) of a functionally active AgRP peptide [inverse agonist, PDB entry 1HYK (61)], with the amino acid residues 87–132, was docked into the putative inactive state model of MC4R **(A)**. This peptide is stabilized by several disulfide bridges and involved cysteines are represented as spheres. Ligand side-chains participating in receptor interactions are shown as sticks. Of note, in the central active part of this ligand, a _111_*RFF*_113_ motif is located deep in the receptor binding site, whereby arginine 111 is embedded between negatively charged receptor amino acid residues, e.g., D122 and D126. Further significant interactions suggested by our model are (*ligand*/receptor): *F112*/F184, *F113*/F261, *F116*/F284, *Y118*/Y35, *Y109*/Y268, and *R120*/D189. In **(B)**, a surface representation of the agonist α-MSH visualizes the dimension and suggested general localization of α-MSH in the MC4R model, with amino acids constituting the supposed binding site (side chains as green lines) covering the binding pocket. **(C)** The surface representation of AgRP bound to MC4R shows, even a fragmental AgRP, the difference in bulkiness of AgRP compared to the agonistic α-MSH **(C)**.

The MC4R is constituted by structural features typical for GPCRs such as seven transmembrane spanning helices connected by intra- and extracellular loops, an extracellular N-terminus, and an intracellular C-terminal tail (76). Highly conserved amino acids at each helix (Figure 1) are related to common structural properties such as kinks and bulges, or essential intramolecular interactions that are significant for an intrinsically encoded signal transduction competence. However, the MC4R is also characterized by specific putative structural features related to specific amino acid sequence properties. For example, the ECL2 is extremely short with around four amino acids and the highly conserved disulfide bridge among GPCRs between the ECL2 and TMH3 is missing (Figures 1, 2). Moreover, the MC4R is characterized by a regular α-helical conformation of TMH5 because of a methionine instead of a proline (P5.50) that is highly conserved in class A GPCRs and induces a helical kink and bulge. This property is of enormous impact because the orientation of side chains in TMH5 toward the membrane or the transmembrane core depends on this helical feature, which affects properties of the ligand-binding region between the helical core and the extracellular loops (Figures 3, 4).

The highly conserved class A GPCR amino acid motif _7.49_NPxxY in TMH7 is a _7.49_DPxxY motif in the MC4R. To date, approximately 20% of ~300 class A GPCRs (with the exception of olfactory class A GPCRs) are characterized by an aspartate at position 7.49 instead of an asparagine (72%). This sequence variation is not rare, whereby it is not yet clearly defined which structural and functional consequences this evolutionary modification can be attributed to Katritch et al. (79). Based on several mutagenesis and signaling studies in diverse GPCRs [e.g., (80)] or determined structures (81), an aspartate at position 7.49 (negatively charged side chain) has been discussed as potentially contributing to the binding of positively charged sodium ions (increased binding, different spatial coordination) in an interaction network with water molecules and the highly conserved aspartate D2.50 in helix 2 (and other residues of the potential sodium binding site) (45). This suggestion is also supported by the previously determined crystal structure of human protease-activated receptor 1 (PAR1) (82), which includes aspartate at this position. In P2Y12, another class A GPCR with a determined structure (83), endogenous aspartate D7.49 was substituted with an asparagine to stabilize the protein sample for crystallization. Of note, the PAR1 crystal structure presents a sodium binding site that differs to other crystallized GPCRs [e.g., in the high resolution structure of the adenosine-receptor A_2A_R (84)], whereby the location of sodium is differently adjusted between positions D2.50 and D7.49. The PAR1 structure supposes that a negatively charged side chain leads to closer interaction with the sodium ion toward D7.49 in contrast to GPCRs with a non-charged asparagine side chain at this position, with the sodium ion located between D2.50 and N7.45 [reviewed in Katritch et al. (79)]. It was also speculated that a double aspartate constellation (D2.50/D7.49), as observed in all MCRs and in the P2Y12 orthologs (85), should bind divalent cations instead of receptors with a more conserved D2.50/N7.49 variation. In addition, a D7.49 should alternatively support binding of a sodium ion in GPCRs with an N2.50 [reviewed in Katritch et al. (79)]; however, this cannot be the case for the MC4R with a D2.50 (45).

For the hMC4R, previous mutagenesis studies showed a fully hampered signaling capacity for the D298A (D7.49A) substitution, supporting a fundamental role of this position in receptor signaling (whereby ligand binding was not significantly affected) (63). Interestingly, the endogenous D298N variant in pig MC4R is controversially discussed to be involved in weight gain [reviewed in Switonski et al. (86)]. A previous comparison between the D7.49N variant in pig MC4R and hMC4R have not pointed toward differences in resulting binding or signaling capacities at both receptors and have not provided any significant impact of this variant on ligand binding or cAMP signaling. In summary, the MC4R and other MCRs exhibit a significant variant in this conserved TMH7 motif, which may impact the binding of ions in class A GPCRs. The sodium-ion and surrounding water molecules are in tight hydrogen bond interactions with surrounding hydrophilic amino acid side chains and may impact signaling properties, including signaling pathway preferences, TMH7 orientation, and receptor dynamics, implying regulation of transitions between different receptor activity states (79, 81). Of note, in all available active state GPCR structures, a sodium ion is absent, which supports specifically a role in the constitution of a signaling-competent conformation. In conclusion, this feature of MC4R might be directly related to signaling properties; however, the detailed mechanism or concrete structural insights are still missing.

In addition, a supposed disulfide bridge in ECL3 (C271–C277, Figure 1) was previously confirmed by mutagenesis studies (57). This disulfide bridge is in accordance with findings at the MC2R (87). Moreover, a second extracellular disulfide bridge should be postulated between a further cysteine in the ECL3 (C279) and in the transition between the N-terminus and TMH1 (C40). However, mutagenesis studies at these two cysteine residues did not reveal a significant functional influence; therefore, experimental evidence for this supposed disulfide bridge is not yet available. Previous studies suggested that a fragment of the N-terminus should be embedded between the transmembrane helices and act as an intramolecular tethered (partial) agonist (77, 78). This interesting suggestion is not included in the putative MC4R models in the present study, because most naturally-occurring substitutions in the N-terminus region were not yet found to be of functional significance (Supplementary Table 1), which does not support a fundamental role of the N-terminus in signaling regulation. Conservation of the MC4R N-terminus among receptor orthologous is, however, high, even at the HxWNRS motif (Supplementary Figure 2), which supposedly interacts intermolecularly with amino acids in the helical bundle, thereby contributing to the signaling capability of the receptor (permanent basal activity). However, at this motif, several species-specific variations can be observed, e.g., several fish MC4Rs expose an xY/FRNHS motif, which deviates significantly from the corresponding human MC4R N-terminus.

Based on molecular dynamic simulations, a recent study reported the potential involvement of the N-terminus in the stepwise binding of a small molecule (antagonist MCL0129) and that ligand selectivity parameters are also potentially encrypted in the N-terminus (52). Notably, the N-terminal sequence is not conserved among the different MCR subtypes (Figure 1). Finally, it can be supposed that the MC4R N-terminus can contribute to receptor signaling properties; however, further experimental studies are required.

### Ligand Binding at the MC4R

The different MSH variants are agonistic peptide ligands that bind to the MC4R. Moreover, the endogenous inverse agonist AgRP inhibits basal constitutive signaling. The mechanism of ligand binding and ligand selectivity are important aspects required for gaining a comprehensive understanding of this receptor from biological, medical, and pharmaceutical perspectives. This is further complicated by the assumption that several accompanying processes or factors contribute to ligand binding, such as the oligomeric MC4R constellation (88), or zinc ions (89). However, in regard also to developed non-endogenous MC4R ligands (9, 29, 35), this issue requires investigation. Therefore, in the following section, we briefly present and discuss insights into ligand binding with particular emphasis on structural aspects.

In our recent study, we presumed particular binding modes of the agonistic peptides α-MSH (linear) and the cyclic setmelanotide (also termed RM-493) at the MC4R (50). Briefly, a high number of intermolecular contacts with the transmembrane region (TMHs 2-7) as well as ECL2 and ECL3 determines the mode of α-MSH binding, which is generally in accordance to putative ligand/receptor contacts [reviewed in Ericson et al. (9) and Tao (16)]. Proposed direct substantial contacts are, for example (α*-MSH*/MC4R amino acid denotation), *W9*/F261; *R8*/E100, D122, D126; *F7*/F184; and *H6*/Y268. In α-MSH and other MC4R ligands (e.g., setmelanotide), the central recognition motif _6_HFRW_9_ (9) is located between the extracellular ends of the transmembrane helices (Figure 3). However, in particular, the N-terminal histidine of both ligands differs in its orientation. The H4 in RM-493 is most likely located between TMH3 (N123) and TMH4 (F184); however, in the α-MSH/MC4R complex, the corresponding H6 is located between TMH6 (Y268) and TMH5 (S191). Finally, it can be postulated that both ligands share specific interactions of the highly conserved ligand motif HFRW with MC4R [see also (90)]. Yet, specific amino acids such as N-terminal histidine residues differ in their detailed interactions with side chains in MC4R (50), which is likely justified by the different linear or cyclic structures of both agonists.

Based on kinetic ligand binding studies, two different tandemly arranged and presumably interrelated ligand binding sites in oligomeric MC4R complexes were suggested (88), which are also associated and complementary to studies on MC4R oligomers (42–44) (see chapter MC4R oligomerization—putative protomer interfaces and contribution to signaling) and bivalent ligand binding (presented below in detail) (37–41). In addition, zinc ions can act agonistically and as positive allosteric modulators in the MC4R (89).

These insights into agonistic or allosteric ligand binding generate interest regarding differences in the binding of agonists in comparison to the inverse agonistic ligand AgRP [for Gs-mediated cAMP signaling, see (49)]. The available solved structure of a functionally active AgRP peptide [PDB entry 1HYK (61)] was docked into the MC4R model (Figure 4). In the central part of this ligand, a _111_RFF_113_ motif is located deep in the receptor, whereby R111 is surrounded by the negatively charged, receptor amino acid residues E100, D122, and D126. This presumable interaction corresponds to experimental hints generated in different studies on AgRP binding (63–66). Further significant interactions suggested by our model are (*AgRP*/MC4R amino acid denotation) namely, *F112*/F184, *F113*/F261, *F116*/F284, *Y109*/Y268, *Y118*/Y35, or *R120*/D189. These potential ligand-receptor interactions are likely AgRP-specific and not relevant for the binding of the agonistic ligands MSH or setmelanotide (50). The AgRP contacts are generally spread over diverse receptor parts, including transmembrane helices, the extracellular loops, and the N-terminus. This may help explain the antagonistic and, especially, the inverse agonistic effects of AgRP, whereby these contacts may function as constraints keeping the MC4R in a practically inactive state conformation, at least for certain signaling pathways (49).

Of note, the C-terminal sequence of AgRP is highly conserved among different species and is characterized by a defined patterned distribution of cysteine residues and disulfide bridges (Supplementary Figure 3), while the N-terminal half is not highly conserved among many orthologs [this structural part is not included in the crystal structure [PDB entry 1HYK (61)]. AgRP is cleaved intracellularly by a convertase at position 83 (91). Accordingly, in our putative binding mode for AgRP at the MC4R (Figure 4), the conserved region contributes exclusively to intermolecular contacts.

**Figure 5:**
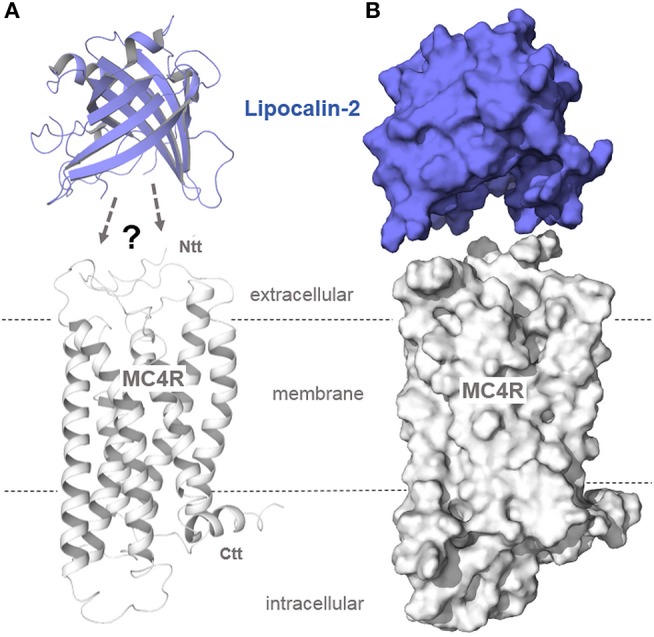
Structural protein dimensions of MC4R and lipocalin-2. In a recent study (33), lipocalin-2 (LCN2), a bone-derived hormone with metabolic regulatory effects, was reported to bind to MC4R, thereby suppressing appetite (anorexigenic effect). Without detailed experimental studies on the binding mechanism between MC4R and the β-barrel folded lipocalin, predictions on how this hormone interacts with the receptor cannot be made. However, comparing the structural dimensions of the engineered lipocalin variant Anticalin US7 [PDB entry 4MVI (92)] with our MC4R model (**A**, as a ribbon presentation; **B**, as a surface representation) shows the huge hormone protein that most likely interacts with the MC4R in a manner completely different to other peptidic MC4R ligands (Figures 3–4).

A recent study reported that lipocalin-2, a bone-derived protein, acts as an agonist at the MC4R, whereby lipocalin-2 crosses the blood-brain barrier and interacts with the MC4R in the hypothalamus (33). The crystal structure of lipocalin-2 is shown in Figure 5. Additionally determined apo-lipocalin or lipocalin-complex structures (e.g., PDB entries 1KXO, 1LKE, and 1N0S) should be used to estimate the putative binding sites of this protein for interaction with the MC4R. As depicted in Figure 5, only the spatial dimensions of both interacting proteins can be visualized, reasoned by the yet absent functional-molecular data concerning the putative lipocalin binding mechanism to this receptor.

Interestingly, a previous study reported that zfMC4R shows increased ACTH sensitivity mediated by the escort protein MRAP2a (93). Similar results with MRAP2 were reported recently for chicken (60) and for the human MC4R (71). Only MC2R has been known to usually interact with ACTH with high affinity. The question that arises is: how can this effect and difference to other MCRs be explained? To obtain structural insights into similarities or differences between zfMC4R/MSH or zfMC2R/ACTH complexes, we designed zfMC4R-αMSH and zfMC2R-ACTH models. These models may help to generate hypotheses concerning the reported effect of MRAP2a in interplay with zfMC4R, leading to increased ACTH-ligand sensitivity (93).

**Figure 6:**
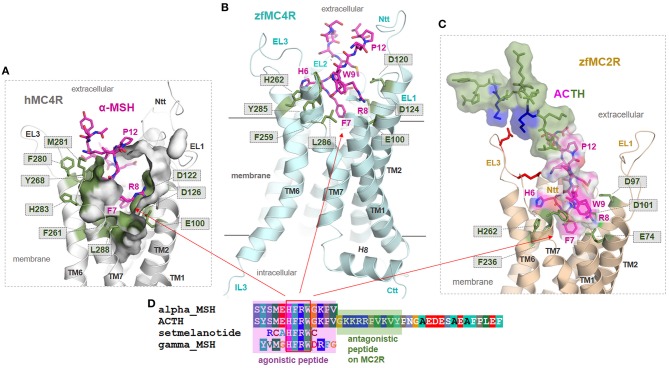
Structural insights into ligand binding at human and zebrafish MCR subtypes. **(A)** The human hMC4R/α-MSH complex model (inner clipped surface representation of the binding pocket) assumes that the agonist (magenta) binds into a pocket-like cleft between the extracellular loops (EL) and the transmembrane helices (TM). Specific conserved amino acids (Figure 3) covering the ligand binding pocket (green sticks). Importantly, in α-MSH and other MC4R ligands, a central amino acid motif _6_HFRW_9_ is essentially involved in ligand recognition and induction of ligand effects (9). **(B)** This agonistic motif is likely located between the helices also in the zfMC4R and interacts with the conserved amino acids among MCRs. Consequently, we assume comparable MSH binding modes in hMC4R and zfMC4R. **(C,D)** The zfMC2R (backbone representation) most likely binds the N-terminal MSH-like part of ACTH (translucent surface and amino acids as sticks) similar to MSH in MC4Rs; however, interplays differently with the extended ligand part that acts antagonistically if supplemented as a separated peptide. Here, several positively charged amino acid residues are allocated and should interact with the extracellular loops, e.g., negatively charged amino acids (red sticks) in the ECL3.

First, highly essential residues involved in ligand binding at the hMC4R are observed to also be conserved in zfMC4R, yet are also located in corresponding positions of the zfMC2R (Figure 6, Supplementary Figure 1). This circumstance helps to explain why these two receptors are able to bind αMSH or the MSH amino acid moiety of ACTH (Figure 6), in the case of zfMC4R through MRAP2a participation, respectively. These important amino acids for MSH binding include (hMC4R numbering): E100, D122, D126, F261, H264, and F284 (Figure 6). This conservation suggests that the binding mode, at least in the central core of MSH (_6_HFRW_9_), is most likely comparable between human and zebrafish MC4R and that the N-terminal MSH-like moiety in ACTH is also bound to zfMC2R in a similar binding mode as suggested for αMSH in the MC4Rs. This is further supported by pathogenic findings, where an essential amino acid of the N-terminal ACTH peptide (Arg8Cys) is mutated, causing glucocorticoid deficiency by decreased hormone binding (94). However, the difference between ACTH and MSH binding at MCRs is likely associated with the amino acids 13–24 of ACTH. They can interact with the receptor at several structural parts external of the MSH binding site, especially with the extracellular loops. These additional interactions between ACTH and zfMC2R, e.g., with the ECL3 (Figure 6), may be responsible for the endogenous capacity of MC2R to interact with ACTH. Finally, based on these models, it can be postulated that MC4Rs and other MCRs might be characterized by structural features, e.g., a longer N-terminus compared to MC2R (Figure 1), that excludes or hinders steric and/or biophysical ACTH binding. This would precipitate the suggestion that MRAP2 interaction with MC4R must modify specific structural features such as the N-terminus (see also Supplementary Figure 1), which differs compared to MC2R to enable high ACTH affinity binding. Detailed experimental studies addressing this question have not yet been performed.

### MC4R Oligomerization—Putative Protomer Interfaces and Contribution to Signaling

The MC4R is known to constitute homodimers or homo-oligomers (42–44). In addition, heteromeric MC4R interrelations with GPR7 (47) or MC3R (40) were reported recently. It remains elusive how these oligomeric protein constellations, if functionally relevant, are arranged and what their functional consequences regarding receptor signaling properties are.

Generally, GPCR dimerization has been found to impact physiological aspects (95–97) and pathophysiological conditions (98–101). Oligomerization of GPCRs is known to potentially influence ligand binding (102, 103), G-protein selectivity (104), signal transduction mechanisms (105, 106), or cell surface expression (107). Moreover, allosteric effects between individual protomers were reported (108). Recently, forced MC4R monomerization was revealed to cause almost twice the maximum in cAMP-signaling compared to the MC4R with the capacity to constitute oligomers (72). This leads to the assumption that signaling properties of MC4R monomers and oligomers differ and that MC4R/G-protein stoichiometry depends on this feature, as discussed for several GPCRs (109). The aforementioned conclusion is generally in accordance with the suggestion of two different tandemly arranged ligand binding sites in oligomeric MC4R complexes (88). In relation to this finding, it must be noted that GPCRs may, in fact, be expressed as a mixture of monomers and homo-oligomers and that the different forms interconvert dynamically (110). Consequently, GPCRs might be expressed in specific monomer/homodimer ratios at the membrane and in intracellular compartments (111–113). In such cases, the respective contributions of monomers and dimers to the cellular signaling output are usually unclear, which hinders appropriate interpretation of, e.g., dose-response curves in terms of a dissection between monomer/oligomer mediated signaling. The potential functional relevance of oligomeric constellations is also confirmed for the zfMC5R, whereby receptor oligomerization is hindered by the protein interaction with zebrafish Mrap2 (114).

**Figure 7:**
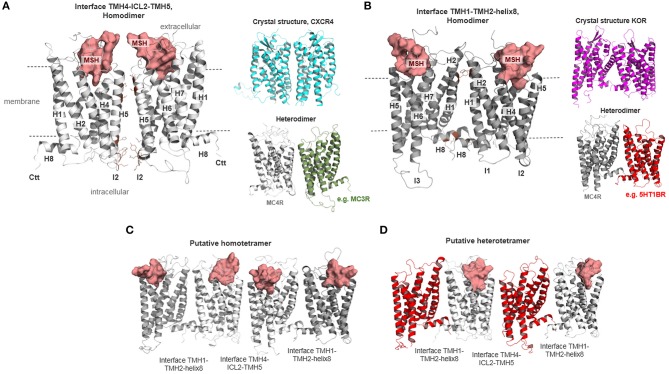
Different putative MC4R homodimer and heteromer arrangements. The MC4R has the capacity to constitute homodimers or homo-oligomers (42–44). In **(A,B)**, two different homodimeric MC4R constellations are supposed with different interfaces. **(A)** MC4R homodimer with an interface between parts of TMH4 (or H4), intracellular loop 2 (ICL2 or I2), and the TMH5 (or H5) based on the determined dimeric crystal structure of CXCR4 (cyan). Such arrangements should also be assumed for a heterodimeric constellation as reported for MC4R and MC3R (40) (gray, MC4R; green, MC3R). In **(B)**, the MC4R protomers are in contact via the interface between TMH1-TMH2-H8 based on a determined KOR structure (magenta backbone). Heteromeric MC4R constellations, such as with the GPR7 (47), may also be arranged in such a constellation. Moreover, in **(C)** a tetrameric MC4R arrangement is shown, with the supposed interfaces alternating between dimers. **(D)** An alternating dimer arrangement for higher complexes is also feasible for tetrameric heteromers.

The next question to address is how MC4R oligomers should be constituted from a structural perspective. Based on previously determined crystal structures of class A GPCR dimers [reviewed in Audet and Bouvier (73)] and many biophysical studies, several oligomeric GPCR protomer arrangements have been suggested (115). Several crystal structures are available, for example, for the chemokine receptor CXCR4 (116), μ-opioid-receptor [MOR (117)], κ-opioid receptor [KOR (118)], opsin (119–122), or the β-adrenergic receptor 1 [β-1AR (123)]. In opsin, KOR, and β-1AR, the protomer interface is located at TMH1-TMH2 and helix 8. Interfaces between GPCR protomers at the region from ICL2 to TMH4 (124–126), TMH4-TMH5 (127), or TMH5-TMH6 (128–130) were also supported by experimental data for different GPCRs. For MC4R, a putative protomer-protomer interface between TMH3-ICL2-TMH4 (Figure 7), respectively, was recently suggested based on experiments leading to a forced MC4R monomerization by modification of specific structural components in this region (72). As other protomer-protomer interfaces cannot be excluded, we suggest further potential homo-/heteromeric MC4R dimer packing constellations based on the afore described information on different GPCRs: (i) an MC4R homodimer with an interface between TMH3-ICL2-TMH4, and (ii) an interface at TMH1-TMH2-helix8 (Figure 7). Such dimer packing constellations in an alternating arrangement can be also assumed for higher order complexes such as tetrameric oligomers.

The question to be asked here is whether this structural information is relevant for receptor function beyond the MC4R/G-protein stoichiometry (109) or the interplay between two ligand binding sites (88). New developments of potent so-called homobivalent ligands, differing in the length and nature of the spacer between two pharmacophore sites, supported the notion of the functional relevance of receptor dimers or higher oligomers. An example of a relationship between structural properties, oligomeric arrangements, and molecular biology is the recently described MC4R homobivalent agonist CJL-1-87 with a 20-atom spanning spacer connecting two “HdFRW” ligand motif pharmacophore moieties (core amino acids of MSH, Supplementary Figure 4) (39, 40). The unique pharmacology of this homobivalent ligand with increased *in vitro* binding affinity was suggested to enhance *in vivo* functional potency and increased ligand residence time has a crucial impact on the ligand's effectiveness. This results in decreased food intake in rodents after fasting and might be a result of interaction either with MC3R homodimers, MC4R homodimers, or heterodimers between MC3R and MC4R. Based on our dimeric homology models (Supplementary Figure 4), we show that in a putative MC4R homodimer with a TMH4/5-TMH4/5 interface (Figure 7A), the two ligand moieties can be located in the orthosteric (MSH) binding sites at both protomers, whereby a distance of approximately 25 Å between the ligand moieties would be ideal for the specific linker length as reported. In case of an alternative TMH1/TMH1-helix 8/helix 8 protomer interface (Figure 7B), the approximate distance to be bridged by the linker would be above 55 Å and hence a bivalent ligand molecule would not bind into both protomers of this dimer. Based on these rational structural perspectives, the design of appropriate ligand constructs with specific properties should be refined and potentially improved in further approaches of directed ligand design.

### Mechanisms and Structural Features Involved in Signaling Regulation at the MC4R

**Figure 8:**
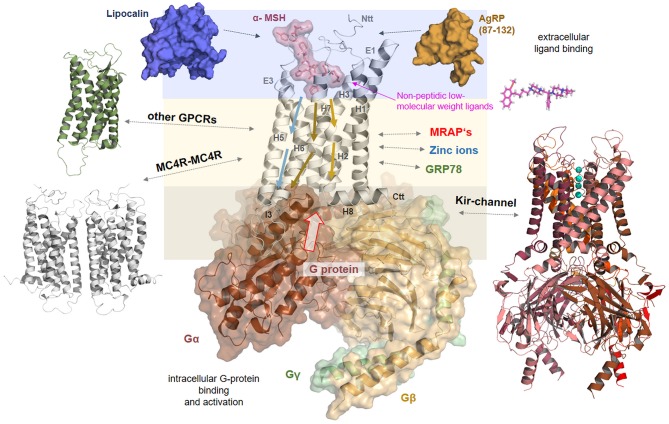
Signaling regulation and putative interaction with tuning factors at the MC4R. This complex model between MC4R, α-MSH, and Gs visualizes different steps in signal transduction that are interrelated. Starting from the extracellular site, the ligand binds between the extracellular loops (E1-3) and transmembrane helices (H1-7). Ligand binding induces structural rearrangements in the transmembrane spanning part. It can be postulated that biased agonism is also associated with diversely initiated signaling pathways in the transmembrane core (colored arrows). The G-protein can fit into the receptor open crevice at the intracellular site, which is accessible in consequence to the ligand-induced receptor activation. This ternary complex finally stabilizes an active state conformation of the receptor with a defined affinity and selectivity for different intracellular effectors. In addition, the melanocortin system can be assisted by the membrane spanning dimeric (131) melanocortin receptor accessory protein (MRAP2) (132). Homo- or hetero-oligomerization of the MC4R (Figure 7) is a further regulating element in the signaling process (48). MC4R also directly or indirectly impacts the ion channel Kir 7.1 (21), is influenced by interaction with GRP78 (133), or zinc and sodium ions (52, 89). Based on a recent study (52), it can be supposed that small drug-like molecules bind into an allosteric binding site between the transmembrane helices under active support of amino acid residues at N-terminal extension.

The above-described information regarding the MC4R amino acid sequence, potential MC4R structure, available valuable insights into ligand binding, and associated receptor regulation mechanisms such as oligomerization or interaction with other proteins, lead to the key question of how the signaling process at the MC4R occurs in detail. Therefore, we summarized available information in a simplistic scheme (Figure 8).

In brief, signal transduction at the MC4R generally includes: *(a)* extracellular binding of ligands such as MSH for signal induction or in the case of AgRP to inhibit agonist binding and decrease basal signaling activity, *(b)* signal transduction through the transmembrane spanning region by movement of specific helical parts, and *(c)* to finally enable specific binding and activation of intracellular transducers/effectors such as G-proteins, arrestin. These general steps are concerted and are strongly dependent on structural features.

In detail, at the extracellular site, the ligand binds at specific determinants between the ECLs and the TMHs (via complementary recognition motifs, Figures 3, 4), inducing structural rearrangements in the transmembrane spanning region, specifically at helices 5, 6, and 7. This leads to a shift of the receptor structure toward an activity state that is at an energetically higher predisposition to bind intracellular signal transducers. Ionic interactions such as with zinc (89) or sodium (52) may impact the dynamic process of ligand binding or conformational changes. Moreover, the oligomeric MC4R arrangement may influence ligand binding properties by the mutual impact of the interacting protomers, respectively (88). Of note, it can be postulated that biased signaling of specific MC4R ligands is associated with diversely initiated signaling pathways in the transmembrane region, as has been shown for MSH vs. setmelanotide ligand effects, which induces different selective favoring pathways (50).

However, after ligand binding and modifications in the transmembrane region, the G-protein molecule can fit spatially into a particularly defined receptor crevice at the intracellular site, which also exposes complementary determinants responsiveness to certain interacting molecules such as G-proteins or arrestin receptor selectivity at the intracellular site as suggested for, e.g., rhodopsin (134). This ligand/receptor/G-protein (or arrestin) ternary complex finally stabilizes an active state conformation of the receptor. As described above, this process can be assisted or modified by further membrane spanning interaction partners such as the MC4R itself in homomeric constellations, through interaction with other GPCRs in heteromers, or proteins such as MRAP2 (132), or KIR channels (21). It must be noted that in consequence of these functionally and structurally distinguishable levels of signal transduction (Figure 8), the resulting signal [e.g., (135, 136)] can be specified or tuned at certain intermediate states, such as during selective ligand, ion, or G-protein binding. The exact deciphering of hot-spots in the receptor/ligand interplay, enabling a pathway selective signaling modulation, poses a challenge. Finally, these general and detailed insights into MC4R signaling enable a more detailed reflection of putative pathogenic mechanisms at the protein level.

### Naturally Occurring MC4R Mutations in a Structural-Functional Context

**Figure 9:**
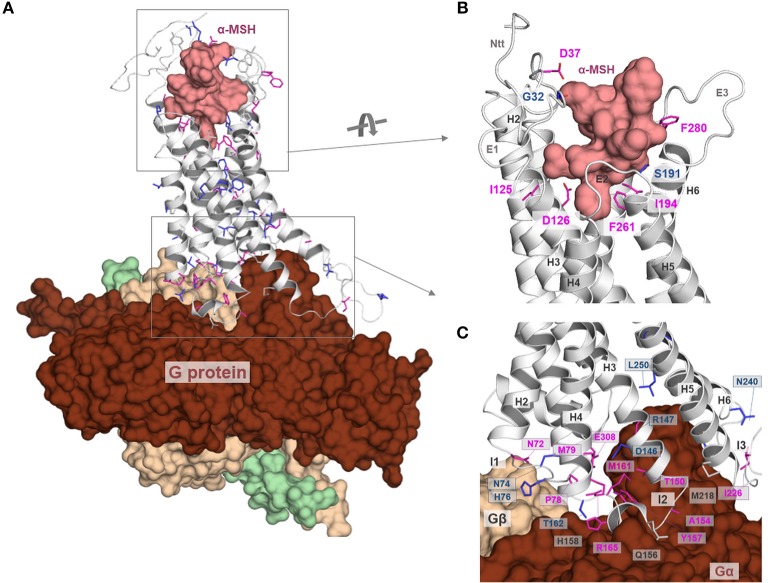
Structural mapping of pathogenic single side chain substitutions in MC4R. **(A)** Positions of naturally-occurring side chain substitutions (Supplementary Table 1) are highlighted on a putative active state MC4R model in interaction with α-MSH and G-protein. This structural mapping enables a detailed estimation of individual molecular mechanisms for each mutation by evaluation of the wild-type amino acid function. The ligand and the heterotrimeric G-protein (colors: α-subunit, chocolate: β-subunit, wheat; γ-subunit green) are shown as surfaces; the receptor is presented as a backbone ribbon (gray). Residues associated with pathogenic substitutions are highlighted (sticks), whereby the color magenta indicates an experimentally proven reduction in signaling and the color blue a reported signaling property that corresponds to wild-type (“like wild-type,” Supplementary Table 1) (no functional molecular data reported so far, white). **(B)** This figure shows α-MSH in interaction with MC4R as suggested by our docking studies (Figure 3) and highlights exclusively those wild-type amino acid residues of reported pathogenic substitutions that are in close proximity or in direct interacting contact to α-MSH. In conclusion, these residues are potentially involved in ligand binding and any side chain variation may lead to a modified ligand binding capacity. Potential hydrogen bonds between α-MSH and MC4R are shown as dashed lines (black). **(C)** Similar to **(B)**, also the detailed MC4R/G-protein interface can be analyzed on the potential participation of wild-type residues that might be involved in G-protein binding. Indeed, the model suggests that several residues are in direct interaction distance to the α-subunit of Gs, or, e.g., H76 in close proximity to the β-subunit. In conclusion, pathogenic mutations at these positions can be suggested to modify the MC4R/Gs interface, which, in conclusion, leads to a reduced signaling capacity for the respective pathway.

As previously mentioned, inactivating MC4R mutations are the most frequent monogenic cause of obesity (24). In the Supplementary Table 1, we summarized reported naturally occurring MC4R single side-chain amino acid substitutions (stop mutations, deletions, and double substitutions were excluded) and mapped their positions on the α-MSH/MC4R/G-protein ternary complex model (Figure 9) to estimate general and detailed molecular features of individual substitutions. Moreover, we also examined each study for experimental data characterizing the respective MC4R variant by *in vitro* experiments to roughly evaluate functional parameters influenced by these respective mutations.

One hundred and sixty-five different substitutions at 129 distinct MC4R amino acid positions were collected; hence, more than one-third of all MC4R residues are exchanged in obese patients (Supplementary Table 2). First, it must be emphasized that for 24 of the 165 MC4R variants collected, there is no functional *in vitro* data (Supplementary Tables 1, 2, Supplementary Figure 5). Of specific note, a receptor function “like wild-type” with either respect to cAMP accumulation and/or α-MSH binding were characterized *in vitro* in 71 of the reported mutations (Supplementary Table 1, Supplementary Figure 5). In contrast, 92 of the 165 mutants were reported to cause a decrease in α-MSH binding and/or cAMP accumulation or cell surface expression (22 mutants were reported to be contradictory with both like wild-type or decreased signaling). In conclusion, aside from a huge amount of reports on pathogenic MC4R variations, this analysis also shows the huge gap of information that we are still faced with, particularly concerning the important question: What is the molecular relationship (e.g., increased, decreased, or selective signaling pathways) between an identified sequence variation and a dysfunction in obese patients? This further leads to the question concerning how to relate an identified MC4R variant causatively with a certain specific phenotype if the *in vitro* characterization does not reveal drastic changes in specific molecular aspects such as cell surface expression or G-protein activation.

Of note, in addition to the Gs-mediated cAMP signaling pathway, MC4R has been shown to regulate signaling using Gi/Go (137), Gq (135), c-Jun N-terminal kinase (JNK) (138), ERK1/2 signaling (139), and cAMP-independent activation of the ion channel Kir 7.1 (21). Altogether, characterizing identified naturally-occurring MC4R variants systematically with respect to different signaling pathways would be of interest and may support the identification of the exact pathogenic mechanism underlying the patient-phenotype, as shown recently (19, 50). The afore described information makes it reasonable to postulate that contemporary approaches of MC4R mutant investigation should take a more completed set of signaling pathways under consideration, especially for mutations that have not shown any effects in Gs-mediated signaling (highlighted in Supplementary Table 1 with “=”). Several mutations have been identified to induce biased preferred signaling in regards to ERK1/2 and Gs-mediated cAMP signaling (139) (marked with “^*^” in Supplementary Table 1).

Second, inactivation of a functional ternary complex constituted by ligand/receptor/intracellular signal transducer can occur at different levels but generally, result in the “inactivation” of the complex unit. Receptor mutations can impair protein function at the transcription, translation, folding, ligand binding, or downstream signaling level (140). Impaired translation and protein misfolding can result in a reduced number of receptors presented at the cell surface. Changes of signaling may be related to modifications in the ligand binding properties or in the MC4R/G-protein contact interface. Based on our combined mutant collection and three-dimensional mapping of naturally-occurring MC4R mutations, we are able to provide an analysis of substitutions that either modulate (i) ligand binding, (ii) signal transduction in the transmembrane domain, or influence the (iii) receptor/G-protein interplay.

In principle, the overall distribution of all reported naturally occurring substitutions is spread over the entire receptor (Figure 9A, Supplementary Table 1). To analyze the reported MC4R variants in regard to their respective structural-functional relationships, we here describe several examples related to either ligand binding, G-protein recognition, or located at well-known micro-switches or micro-domains (141) in the helical bundle involved in signal transduction through the membrane.

#### The Ligand Binding Region

Interestingly, 21 naturally occurring single side-chain substitutions in the N-terminal domain at 14 different positions show predominantly no impact on ligand binding or cAMP signaling (Supplementary Table 1). This high ratio of reported naturally-occurring substitutions without a significant change in receptor/G-protein coupling suggests that the N-terminus may not contribute significantly to α-MSH binding or Gs-mediated signaling. Therefore, these variants may impact further receptor signaling pathways or functions, which should be investigated in extended experimental studies.

However, the putative binding sites of MC4R ligands were described recently in detail (50, 52) and in the sections above (Figures 3, 4). We extracted the following amino acid positions of reported pathogenic mutations as located in the sensitive region responsive for binding contacts to MC4R ligands: G32, D37, I125, D126, S191, I194, F261, and F280 (Figure 9B). Mutations at these positions may directly prevent or modulate appropriate ligand binding, which is the case for both agonistic and antagonistic ligands. This might be due to local structural changes in the ligand entrance environment of the receptor, thereby preventing a spatial “fit-in” of the ligand. It can also cause direct changes within the ligand binding pocket corresponding to different structural/chemical interaction patterns between the receptor and the ligand as suggested for the wild-type MC4R. This concerns, in particular, main interactions such as those observed, e.g., with the MC4R residue D126 (substitution D-Y) or for mutants at F261 (substitution F-S) (see Supplementary Table 1).

#### The Membrane Spanning Region

Our structural mapping of pathogenic mutations highlights a clustering of variations at the TMH1/2/7 interface in the transmembrane region. These include P299H, I301T, A303T, R305W/S in TMH7; E61K, N62S in TMH1, and A87D and D90N in TMH2. Notably, in TMH1, signal downregulating substitutions were identified in an extended amino acid stretch from L54 to N62. Similarly, several mutations in TMH2 between A87–N97 are clustered, suggesting that the TMH1-TMH2 interface is of particularly high importance for receptor-misfolding and/or -mediated signal transduction. This assumption is supported by the D90N^2.50^ mutation that has been identified in several independent studies of obese cohorts (42, 142–144). The conserved wild-type residue D90 is suggested to be essential for water-related activation by allosteric sodium binding (45, 79). These examples of pathogenic mutants in the transmembrane region are significant because they are located in structural micro-domains involved either in keeping the receptor in a structural conformation that is predisposed to become activated by agonistic ligands, or they are at positions (e.g., in TMH7) important for stabilizing the active state conformation after ligand interaction. In any scenario, the interruption of either hydrophilic or hydrophobic interactions in this area leads to a receptor state that is unable to become (fully) activated by ligand action.

#### The G-Protein Binding Site

Comparable to the disturbed ligand recognition, several pathogenic mutations can be attributed directly or indirectly to negatively modulate the G-protein/receptor interplay. For example, five of eight residues of the MC4R ICL1 have been reported with pathogenic effects (Supplementary Table 2), definitively indicating the high impact of this intracellular loop for MC4R functions. Our complex homology model (Figure 9C) supports a potential interaction between ICL1 and the Gβ subunit of Gs (via hydrogen bonding from the ICL1 amino acids H76 and K73). In accordance with the occurrence of pathogenic MC4R variations in this receptor part and our ternary complex model, the recently published structure of a class B GPCR (GLP1R) as a complex with a trimeric Gs-protein has shown detailed structural information regarding an ICL1-Gβ interplay (145). This is further supported by experimental findings at the μ-opioid receptor (MOR), whereby initial interactions between the G-protein and ICL1 may be involved in G-protein coupling specificity (146).

Generally, the ICL2 and ICL3 of GPCRs are both well-known to interact with the Gα subunit of Gs (54, 147, 148), hence mutations in this region are expected for most GPCRs to directly affect G-protein activation. Based on our MC4R models, the ICL2 consists of 13 residues, of which six have been reported to be a pathogenic mutation. Three of these mutations have been shown experimentally to reduce cAMP signaling (A154D, Y157S, M161T) (Supplementary Table 2). In remarkable contrast, substitution H158R has been reported to result in a 6-fold increased basal activity, which characterizes this mutation as a constitutively activating mutation (CAM) (149). In addition, seven pathogenic MC4R mutations at six positions are located in the ICL3; however, only two of these six mutations (Figure 9C) were already characterized as reduced in Gs-mediated signaling (I266T, G238D) (Supplementary Table 1), indicating the need of extended functional characterization to evaluate the causality between these variants and patient phenotypes. Collectively, these mutations and functional insights highlight all three intracellular loops as important for G-protein coupling and activation. As described above, the ICL2 and ICL3 of the MC4R share a high abundance of pathogenic substitutions with low or no impact on Gs-mediated cAMP signaling, which predestinates these structural components as potentially important for other signaling pathways mediated by, e.g., Gq-protein.

## Conclusions

Altogether, the present study provides an analysis of ligand interactions and pathogenic variations using an MC4R model to gain detailed insights into the mode of action and the structural and functional relationships of the MC4R. This included putative 3D conformations and complexes, and associated functional aspects such as details of ligand binding or oligomeric constellations. Of importance for MC4R research are advanced insights into questions regarding biased signaling, signaling modulation by interacting partners or MC4R modulation by designed ligands, including linear or cyclic peptide ligands, synthetic small molecules, or bivalent ligands. MC4R-related research with a primary focus on pharmaceutical, structural, and biochemical aspects is vital, with the potential to study many interesting aspects of class A GPCRs at this receptor.

## Data Availability

All datasets generated for this study are included in the manuscript and/or the Supplementary Files.

## Author Contributions

NH data analysis, modeling studies, figure preparations, wrote the manuscript, and final approval. GK project idea, modeling studies, figure preparations, data analysis, wrote the manuscript, and final approval. MS, DK, DS, LS, JC-R, HK, PK, and HB data analysis, contributions to writing of the manuscript, and final approval. PS project idea and co-ordination, figure preparations, data analysis, wrote the manuscript, and final approval.

### Conflict of Interest Statement

The authors declare that the research was conducted in the absence of any commercial or financial relationships that could be construed as a potential conflict of interest.

## References

[1] Dores RM Londraville RL Prokop J Davis P Dewey N Lesinski N. Molecular evolution of GPCRs: melanocortin/melanocortin receptors. J Mol Endocrinol. (2014) 52:T29–42. 10.1530/JME-14-0050 24868105

[2] Gantz I Miwa H Konda Y Shimoto Y Tashiro T Watson SJ. Molecular cloning, expression, and gene localization of a fourth melanocortin receptor. J Biol Chem. (1993) 268:15174–9. 8392067

[3] Cone RD. Anatomy and regulation of the central melanocortin system. Nat Neurosci. (2005) 8:571–8. 10.1038/nn1455 15856065

[4] Cone RD. Studies on the physiological functions of the melanocortin system. Endocr Rev. (2006) 27:736–49. 10.1210/er.2006-0034 17077189

[5] Tao YX Huang H Wang ZQ Yang F Williams JN Nikiforovich GV. Constitutive activity of neural melanocortin receptors. Methods Enzymol. (2010) 484:267–79. 10.1016/B978-0-12-381298-8.00014-9 21036237

[6] Chen W Kelly MA Opitz-Araya X Thomas RE Low MJ Cone RD. Exocrine gland dysfunction in MC5-R-deficient mice: evidence for coordinated regulation of exocrine gland function by melanocortin peptides. Cell. (1997) 91:789–98. 10.1016/S0092-8674(00)80467-5 9413988

[7] Lute B Jou W Lateef DM Goldgof M Xiao C Pinol RA. Biphasic effect of melanocortin agonists on metabolic rate and body temperature. Cell Metab. (2014) 20:333–45. 10.1016/j.cmet.2014.05.021 24981835 PMC4126889

[8] Simamura E Arikawa T Ikeda T Shimada H Shoji H Masuta H. Melanocortins contribute to sequential differentiation and enucleation of human erythroblasts via melanocortin receptors 1, 2, and 5. PLoS ONE. (2015) 10:e0123232. 10.1371/journal.pone.0123232 25860801 PMC4393082

[9] Ericson MD Lensing CJ Fleming KA Schlasner KN Doering SR Haskell-Luevano C. Bench-top to clinical therapies: a review of melanocortin ligands from 1954 to 2016. Biochim Biophys Acta. (2017) 1863:2414–35. 10.1016/j.bbadis.2017.03.020 28363699 PMC5600687

[10] Rodrigues AR Almeida H Gouveia AM. Intracellular signaling mechanisms of the melanocortin receptors: current state of the art. Cell Mol Life Sci. (2015) 72:1331–45. 10.1007/s00018-014-1800-3 25504085 PMC11113477

[11] Metherell LA Chapple JP Cooray S David A Becker C Ruschendorf F. Mutations in MRAP, encoding a new interacting partner of the ACTH receptor, cause familial glucocorticoid deficiency type 2. Nat Genet. (2005) 37:166–70. 10.1038/ng1501 15654338

[12] Chan LF Metherell LA Clark AJ. Effects of melanocortins on adrenal gland physiology. Eur J Pharmacol. (2011) 660:171–80. 10.1016/j.ejphar.2010.11.041 21211533

[13] Rossi J Balthasar N Olson D Scott M Berglund E Lee CE. Melanocortin-4 receptors expressed by cholinergic neurons regulate energy balance and glucose homeostasis. Cell Metab. (2011) 13:195–204. 10.1016/j.cmet.2011.01.010 21284986 PMC3033043

[14] Krashes MJ Lowell BB Garfield AS. Melanocortin-4 receptor-regulated energy homeostasis. Nat Neurosci. (2016) 19:206–19. 10.1038/nn.4202 26814590 PMC5244821

[15] Van Der Ploeg LH Martin WJ Howard AD Nargund RP Austin CP Guan X. A role for the melanocortin 4 receptor in sexual function. Proc Natl Acad Sci USA. (2002) 99:11381–6. 10.1073/pnas.172378699 12172010 PMC123265

[16] Tao YX. The melanocortin-4 receptor: physiology, pharmacology, and pathophysiology. Endocr Rev. (2010) 31:506–43. 10.1210/er.2009-0037 20190196 PMC3365848

[17] Podyma B Sun H Wilson EA Carlson B Pritikin E Gavrilova O. The stimulatory G protein Gsalpha is required in melanocortin 4 receptor-expressing cells for normal energy balance, thermogenesis, and glucose metabolism. J Biol Chem. (2018) 293:10993–1005. 10.1074/jbc.RA118.003450 29794140 PMC6052205

[18] Li YQ Shrestha Y Pandey M Chen M Kablan A Gavrilova O. G(q/11)alpha and G(s)alpha mediate distinct physiological responses to central melanocortins. J Clin Invest. (2016) 126:40–9. 10.1172/JCI76348 26595811 PMC4701544

[19] Yang LK Tao YX. Biased signaling at neural melanocortin receptors in regulation of energy homeostasis. Biochim Biophys Acta. (2017) 1863(10 Pt A):2486–95. 10.1016/j.bbadis.2017.04.010 28433713 PMC5600658

[20] Agosti F Cordisco Gonzalez S Martinez Damonte V Tolosa MJ Di Siervi N Schioth HB. Melanocortin 4 receptor constitutive activity inhibits L-type voltage-gated calcium channels in neurons. Neuroscience. (2017) 346:102–12. 10.1016/j.neuroscience.2017.01.007 28093215

[21] Ghamari-Langroudi M Digby GJ Sebag JA Millhauser GL Palomino R Matthews R. G-protein-independent coupling of MC4R to Kir7.1 in hypothalamic neurons. Nature. (2015) 520:94–8. 10.1038/nature14051 25600267 PMC4383680

[22] Schaub JW Bruce EB Haskell-Luevano C. Drugs, exercise, and the melanocortin-4 receptor– different means, same ends: treating obesity. Adv Exp Med Biol. (2010) 681:49–60. 10.1007/978-1-4419-6354-3_4 21222259

[23] Collet TH Dubern B Mokrosinski J Connors H Keogh JM Mendes De Oliveira E. Evaluation of a melanocortin-4 receptor (MC4R) agonist (Setmelanotide) in MC4R deficiency. Mol Metab. (2017) 6:1321–9. 10.1016/j.molmet.2017.06.015 29031731 PMC5641599

[24] Farooqi IS Yeo GS O'rahilly S. Binge eating as a phenotype of melanocortin 4 receptor gene mutations. N Engl J Med. (2003) 349:606–9; author reply 606–9. 10.1056/NEJM200308073490615 12908459

[25] Finucane MM Stevens GA Cowan MJ Danaei G Lin JK Paciorek CJ. National, regional, and global trends in body-mass index since 1980: systematic analysis of health examination surveys and epidemiological studies with 960 country-years and 9.1 million participants. Lancet. (2011) 377:557–67. 10.1016/S0140-6736(10)62037-5 21295846 PMC4472365

[26] Dandona P Aljada A Bandyopadhyay A. Inflammation: the link between insulin resistance, obesity, and diabetes. Trends Immunol. (2004) 25:4–7. 10.1016/j.it.2003.10.013 14698276

[27] Lee EC Carpino PA. Melanocortin-4 receptor modulators for the treatment of obesity: a patent analysis (2008–2014). Pharm Pat Anal. (2015) 4:95–107. 10.4155/ppa.15.1 25853469

[28] Todorovic A Haskell-Luevano C. A review of melanocortin receptor small molecule ligands. Peptides. (2005) 26:2026–36. 10.1016/j.peptides.2004.11.024 16051395

[29] Goncalves JPL Palmer D Meldal M. MC4R agonists: structural overview on antiobesity therapeutics. Trends Pharmacol Sci. (2018) 39:402–23. 10.1016/j.tips.2018.01.004 29478721

[30] Bhat SP Sharma A Current drug targets in obesity pharmacotherapy - a review. Curr Drug Targets. (2017) 8:983–93. 10.2174/1389450118666170227153940 28245771

[31] Schonnop L Kleinau G Herrfurth N Volckmar AL Cetindag C Muller A. Decreased melanocortin-4 receptor function conferred by an infrequent variant at the human melanocortin receptor accessory protein 2 gene. Obesity. (2016) 24:1976–82. 10.1002/oby.21576 27474872

[32] Rouault AAJ Srinivasan DK Yin TC Lee AA Sebag JA. Melanocortin receptor accessory proteins (MRAPs): functions in the melanocortin system and beyond. Biochim Biophys Acta. (2017) 1863:2462–67. 10.1016/j.bbadis.2017.05.008 28499989

[33] Mosialou I Shikhel S Liu JM Maurizi A Luo N He Z. MC4R-dependent suppression of appetite by bone-derived lipocalin 2. Nature. (2017) 543:385–90. 10.1038/nature21697 28273060 PMC5975642

[34] Kievit P Halem H Marks DL Dong JZ Glavas MM Sinnayah P. Chronic treatment with a melanocortin-4 receptor agonist causes weight loss, reduces insulin resistance, and improves cardiovascular function in diet-induced obese rhesus macaques. Diabetes. (2013) 62:490–7. 10.2337/db12-0598 23048186 PMC3554387

[35] Chen KY Muniyappa R Abel BS Mullins KP Staker P Brychta RJ. RM-493, a melanocortin-4 receptor (MC4R) agonist, increases resting energy expenditure in obese individuals. J Clin Endocrinol Metab. (2015) 100:1639–45. 10.1210/jc.2014-4024 25675384 PMC4399297

[36] Kuhnen P Clement K Wiegand S Blankenstein O Gottesdiener K Martini LL. Proopiomelanocortin deficiency treated with a melanocortin-4 receptor agonist. N Engl J Med. (2016) 375:240–6. 10.1056/NEJMoa1512693 27468060

[37] Fernandes SM Lee YS Gillies RJ Hruby VJ. Synthesis and evaluation of bivalent ligands for binding to the human melanocortin-4 receptor. Bioorg Med Chem. (2014) 22:6360–5. 10.1016/j.bmc.2014.09.055 25438759 PMC4254589

[38] Lensing CJ Adank DN Doering SR Wilber SL Andreasen A Schaub JW. Ac-Trp-DPhe(p-I)-Arg-Trp-NH2, a 250-fold selective melanocortin-4 receptor (MC4R) antagonist over the melanocortin-3 receptor (MC3R), affects energy homeostasis in male and female mice differently. ACS Chem Neurosci. (2016) 7:1283–91. 10.1021/acschemneuro.6b00156 27385405 PMC5687811

[39] Lensing CJ Freeman KT Schnell SM Adank DN Speth RC Haskell-Luevano C. An *in vitro* and *in vivo* investigation of bivalent ligands that display preferential binding and functional activity for different melanocortin receptor homodimers. J Med Chem. (2016) 59:3112–28. 10.1021/acs.jmedchem.5b01894 26959173 PMC5679017

[40] Lensing CJ Adank DN Wilber SL Freeman KT Schnell SM Speth RC. A direct *in vivo* comparison of the melanocortin monovalent agonist Ac-His-DPhe-Arg-Trp-NH2 versus the bivalent agonist Ac-His-DPhe-Arg-Trp-PEDG20-His-DPhe-Arg-Trp-NH2: a bivalent advantage. ACS Chem Neurosci. (2017) 8:1262–78. 10.1021/acschemneuro.6b00399 28128928 PMC5679024

[41] Lensing CJ Freeman KT Schnell SM Speth RC Zarth AT Haskell-Luevano C. Developing a biased unmatched bivalent ligand (BUmBL) design strategy to target the GPCR homodimer allosteric signaling (cAMP over beta-Arrestin 2 Recruitment) within the melanocortin receptors. J Med Chem. (2018) 62:144–58. 10.1021/acs.jmedchem.8b00238 29669202 PMC6226371

[42] Biebermann H Krude H Elsner A Chubanov V Gudermann T Gruters A. Autosomal-dominant mode of inheritance of a melanocortin-4 receptor mutation in a patient with severe early-onset obesity is due to a dominant-negative effect caused by receptor dimerization. Diabetes. (2003) 52:2984–8. 10.2337/diabetes.52.12.2984 14633860

[43] Elsner A Tarnow P Schaefer M Ambrugger P Krude H Gruters A. MC4R oligomerizes independently of extracellular cysteine residues. Peptides. (2006) 27:372–9. 10.1016/j.peptides.2005.02.027 16289450

[44] Nickolls SA Maki RA. Dimerization of the melanocortin 4 receptor: a study using bioluminescence resonance energy transfer. Peptides. (2006) 27:380–7. 10.1016/j.peptides.2004.12.037 16406142

[45] Tarnow P Rediger A Brumm H Ambrugger P Rettenbacher E Widhalm K. A heterozygous mutation in the third transmembrane domain causes a dominant-negative effect on signalling capability of the MC4R. Obes Facts. (2008) 1:155–62. 10.1159/000138251 20054175 PMC6452123

[46] Kobayashi Y Hamamoto A Takahashi A Saito Y Dimerization of melanocortin receptor 1 (MC1R) and MC5R creates a ligand-dependent signal modulation: potential participation in physiological color change in the flounder. Gen Comp Endocrinol. (2016) 230–1:103–09. 10.1016/j.ygcen.2016.04.008 27080548

[47] Rediger A Tarnow P Bickenbach A Schaefer M Krude H Gruters A. Heterodimerization of hypothalamic G-protein-coupled receptors involved in weight regulation. Obes Facts. (2009) 2:80–6. 10.1159/000209862 20054210 PMC6444828

[48] Kleinau G Müller A Biebermann H. Oligomerization of GPCRs involved in endocrine regulation. J Mol Endocrinol. (2016) 57:R59–80. 10.1530/JME-16-0049 27151573

[49] Yang Z Tao YX. Biased signaling initiated by agouti-related peptide through human melanocortin-3 and−4 receptors. Biochim Biophys Acta. (2016) 1862:1485–94. 10.1016/j.bbadis.2016.05.008 27208795

[50] Clement K Biebermann H Farooqi IS Van Der Ploeg L Wolters B Poitou C. MC4R agonism promotes durable weight loss in patients with leptin receptor deficiency. Nat Med. (2018) 24:551–5. 10.1038/s41591-018-0015-9 29736023

[51] Yang Y Harmon CM. Molecular signatures of human melanocortin receptors for ligand binding and signaling. Biochim Biophys Acta. (2017) 1863:2436–47. 10.1016/j.bbadis.2017.04.025 28478228

[52] Saleh N Kleinau G Heyder N Clark T Hildebrand PW Scheerer P. Binding, thermodynamics, and selectivity of a non-peptide antagonist to the melanocortin-4 receptor. Front Pharmacol. (2018) 9:560. 10.3389/fphar.2018.00560 29910730 PMC5992272

[53] Hanson MA Roth CB Jo E Griffith MT Scott FL Reinhart G. Crystal structure of a lipid G protein-coupled receptor. Science. (2012) 335:851–5. 10.1126/science.1215904 22344443 PMC3338336

[54] Rasmussen SG Devree BT Zou Y Kruse AC Chung KY Kobilka TS. Crystal structure of the beta2 adrenergic receptor-Gs protein complex. Nature. (2011) 477:549–55. 10.1038/nature10361 21772288 PMC3184188

[55] Ballesteros JA Weinstein H Integrated methods for the construction of three-dimensional models and computational probing of structure-function relationships in G-protein coupled receptors. Methods Neurosci. (1995) 25:366–428. 10.1016/S1043-9471(05)80049-7

[56] Hall TA BioEdit: a user-friendly biological sequence alignment editor and analysis program for Windows 95/98/NT. Nucleic Acids Symposium Series. (1999) 41:95–8.

[57] Tarnow P Schoneberg T Krude H Gruters A Biebermann H. Mutationally induced disulfide bond formation within the third extracellular loop causes melanocortin 4 receptor inactivation in patients with obesity. J Biol Chem. (2003) 278:48666–73. 10.1074/jbc.M309941200 14504270

[58] Case DA Pearlman DA Caldwell JW Cheatham TEI Wang J Ross WS AMBER 7. San Francisco: San Francisco University of California (2002).

[59] Wilson KR Todorovic A Proneth B Haskell-Luevano C. Overview of endogenous and synthetic melanocortin peptides. Cell Mol Biol. (2006) 52:3–20. 10.1170/T703 16914082

[60] Sharma S Garfield AS Shah B Kleyn P Ichetovkin I Moeller IH. Current mechanistic and pharmacodynamic understanding of melanocortin-4 receptor activation. Molecules. (2019) 24:E1892. 10.3390/molecules24101892 31100979 PMC6572030

[61] Bolin KA Anderson DJ Trulson JA Thompson DA Wilken J Kent SB. NMR structure of a minimized human agouti related protein prepared by total chemical synthesis. FEBS Lett. (1999) 451:125–31. 10.1016/S0014-5793(99)00553-0 10371151

[62] Haslach EM Schaub JW Haskell-Luevano C. Beta-turn secondary structure and melanocortin ligands. Bioorg Med Chem. (2009) 17:952–8. 10.1016/j.bmc.2008.02.090 18343128 PMC2662475

[63] Yang YK Fong TM Dickinson CJ Mao C Li JY Tota MR. Molecular determinants of ligand binding to the human melanocortin-4 receptor. Biochemistry. (2000) 39:14900–11. 10.1021/bi001684q 11101306

[64] Nickolls SA Cismowski MI Wang X Wolff M Conlon PJ Maki RA. Molecular determinants of melanocortin 4 receptor ligand binding and MC4/MC3 receptor selectivity. J Pharmacol Exp Ther. (2003) 304:1217–27. 10.1124/jpet.102.044974 12604699

[65] Tao Y-X Segaloff DL. Functional characterization of melanocortin-4 receptor mutations associated with childhood obesity. Endocrinology. (2003) 144:4544–51. 10.1210/en.2003-0524 12959994

[66] Chen M Cai M Aprahamian CJ Georgeson KE Hruby V Harmon CM. Contribution of the conserved amino acids of the melanocortin-4 receptor in [corrected] [Nle4,D-Phe7]-alpha-melanocyte-stimulating [corrected] hormone binding and signaling. J Biol Chem. (2007) 282:21712–9. 10.1074/jbc.M702285200 17545153 PMC2704061

[67] Kiefer LL Veal JM Mountjoy KG Wilkison WO. Melanocortin receptor binding determinants in the agouti protein. Biochemistry. (1998) 37:991–7. 10.1021/bi971913h 9454589

[68] Tota MR Smith TS Mao C Macneil T Mosley RT Van Der Ploeg LH. Molecular interaction of Agouti protein and Agouti-related protein with human melanocortin receptors. Biochemistry. (1999) 38:897–904. 10.1021/bi9815602 9893984

[69] Agulleiro MJ Roy S Sanchez E Puchol S Gallo-Payet N Cerda-Reverter JM. Role of melanocortin receptor accessory proteins in the function of zebrafish melanocortin receptor type 2. Mol Cell Endocrinol. (2010) 320:145–52. 10.1016/j.mce.2010.01.032 20138960

[70] Josep Agulleiro M Cortes R Fernandez-Duran B Navarro S Guillot R Meimaridou E. Melanocortin 4 receptor becomes an ACTH receptor by coexpression of melanocortin receptor accessory protein 2. Mol Endocrinol. (2013) 27:1934–45. 10.1210/me.2013-1099 24085819 PMC5427830

[71] Soletto L Hernandez-Balfago S Rocha A Scheerer P Kleinau G Cerda-Reverter JM. Melanocortin receptor accessory protein 2-induced adrenocorticotropic hormone response of human melanocortin 4 receptor. J Endocr Soc. (2019) 3:314–23. 10.1210/js.2018-00370 30652132 PMC6330173

[72] Piechowski CL Rediger A Lagemann C Muhlhaus J Muller A Pratzka J. Inhibition of melanocortin-4 receptor dimerization by substitutions in intracellular loop 2. J Mol Endocrinol. (2013) 51:109–18. 10.1530/JME-13-0061 23674133

[73] Audet M Bouvier M. Restructuring G-protein- coupled receptor activation. Cell. (2012) 151:14–23. 10.1016/j.cell.2012.09.003 23021212

[74] Katritch V Cherezov V Stevens RC. Structure-function of the G protein-coupled receptor superfamily. Annu Rev Pharmacol Toxicol. (2013) 53:531–56. 10.1146/annurev-pharmtox-032112-135923 23140243 PMC3540149

[75] Baltoumas FA Theodoropoulou MC Hamodrakas SJ. Molecular dynamics simulations and structure-based network analysis reveal structural and functional aspects of G-protein coupled receptor dimer interactions. J Comput Aided Mol Des. (2016) 30:489–512. 10.1007/s10822-016-9919-y 27349423

[76] Yang Y. Structure, function and regulation of the melanocortin receptors. Eur J Pharmacol. (2011) 660:125–30. 10.1016/j.ejphar.2010.12.020 21208602 PMC3095696

[77] Srinivasan S Lubrano-Berthelier C Govaerts C Picard F Santiago P Conklin BR. Constitutive activity of the melanocortin-4 receptor is maintained by its N-terminal domain and plays a role in energy homeostasis in humans. J Clin Invest. (2004) 114:1158–64. 10.1172/JCI200421927 15489963 PMC522250

[78] Ersoy BA Pardo L Zhang S Thompson DA Millhauser G Govaerts C. Mechanism of N-terminal modulation of activity at the melanocortin-4 receptor GPCR. Nat Chem Biol. (2012) 8:725–30. 10.1038/nchembio.1008 22729149 PMC3657613

[79] Katritch V Fenalti G Abola EE Roth BL Cherezov V Stevens RC. Allosteric sodium in class A GPCR signaling. Trends Biochem Sci. (2014) 39:233–44. 10.1016/j.tibs.2014.03.002 24767681 PMC4106411

[80] Manivet P Schneider B Smith JC Choi DS Maroteaux L Kellermann O. The serotonin binding site of human and murine 5-HT2B receptors: molecular modeling and site-directed mutagenesis. J Biol Chem. (2002) 277:17170–8. 10.1074/jbc.M200195200 11859080

[81] White KL Eddy MT Gao ZG Han GW Lian T Deary A. Structural connection between activation microswitch and allosteric sodium site in GPCR signaling. Structure. (2018) 26:259–69 e255. 10.1016/j.str.2017.12.013 29395784 PMC5810373

[82] Zhang C Srinivasan Y Arlow DH Fung JJ Palmer D Zheng Y. High-resolution crystal structure of human protease-activated receptor 1. Nature. (2012) 492:387–92. 10.1038/nature11701 23222541 PMC3531875

[83] Zhang K Zhang J Gao ZG Zhang D Zhu L Han GW. Structure of the human P2Y12 receptor in complex with an antithrombotic drug. Nature. (2014) 509:115–8. 10.1038/nature13083 24670650 PMC4174307

[84] Liu W Chun E Thompson AA Chubukov P Xu F Katritch V. Structural basis for allosteric regulation of GPCRs by sodium ions. Science. (2012) 337:232–6. 10.1126/science.1219218 22798613 PMC3399762

[85] Coster M Wittkopf D Kreuchwig A Kleinau G Thor D Krause G. Using ortholog sequence data to predict the functional relevance of mutations in G-protein-coupled receptors. FASEB J. (2012) 26:3273–81. 10.1096/fj.12-203737 22611087

[86] Switonski M Mankowska M Salamon S. Family of melanocortin receptor (MCR) genes in mammals-mutations, polymorphisms and phenotypic effects. J Appl Genet. (2013) 54:461–72. 10.1007/s13353-013-0163-z 23996627 PMC3825561

[87] Yang Y Chen M Kesterson RA Jr Harmon CM. Structural insights into the role of the ACTH receptor cysteine residues on receptor function. Am J Physiol Regul Integr Comp Physiol. (2007) 293:R1120–26. 10.1152/ajpregu.00240.2007 17596328

[88] Kopanchuk S Veiksina S Mutulis F Mutule I Yahorava S Mandrika I. Kinetic evidence for tandemly arranged ligand binding sites in melanocortin 4 receptor complexes. Neurochem Int. (2006) 49:533–42. 10.1016/j.neuint.2006.04.006 16764968

[89] Holst B Elling CE Schwartz TW. Metal ion-mediated agonism and agonist enhancement in melanocortin MC1 and MC4 receptors. J Biol Chem. (2002) 277:47662–70. 10.1074/jbc.M202103200 12244039

[90] Falls BA Zhang Y. Insights into the allosteric mechanism of setmelanotide (RM-493) as a potent and first-in-class melanocortin-4 receptor (MC4R) agonist to treat rare genetic disorders of obesity through an *in silico* approach. ACS Chem Neurosci. (2018). 10:1055–65. 10.1021/acschemneuro.8b00346 30048591 PMC6374207

[91] Creemers JW Pritchard LE Gyte A Le Rouzic P Meulemans S Wardlaw SL. Agouti-related protein is posttranslationally cleaved by proprotein convertase 1 to generate agouti-related protein (AGRP)83-132: interaction between AGRP83-132 and melanocortin receptors cannot be influenced by syndecan-3. Endocrinology. (2006) 147:1621–31. 10.1210/en.2005-1373 16384863

[92] Rauth S Hinz D Borger M Uhrig M Mayhaus M Riemenschneider M. High-affinity Anticalins with aggregation-blocking activity directed against the Alzheimer beta-amyloid peptide. Biochem J. (2016) 473:1563–78. 10.1042/BCJ20160114 27029347 PMC4888463

[93] Agulleiro MJ Sanchez E Leal E Cortes R Fernandez-Duran B Guillot R. Molecular characterization and functional regulation of melanocortin 2 receptor (MC2R) in the sea bass. A putative role in the adaptation to stress. PLoS ONE. (2013) 8:e65450. 10.1371/journal.pone.0065450 23724142 PMC3664627

[94] Samuels ME Gallo-Payet N Pinard S Hasselmann C Magne F Patry L. Bioinactive ACTH causing glucocorticoid deficiency. J Clin Endocrinol Metab. (2013) 98:736–42. 10.1210/jc.2012-3199 23293326

[95] Gassmann M Shaban H Vigot R Sansig G Haller C Barbieri S. Redistribution of GABAB(1) protein and atypical GABAB responses in GABAB(2)-deficient mice. J Neurosci. (2004) 24:6086–97. 10.1523/JNEUROSCI.5635-03.2004 15240800 PMC6729668

[96] Waldhoer M Fong J Jones RM Lunzer MM Sharma SK Kostenis E. A heterodimer-selective agonist shows *in vivo* relevance of G protein-coupled receptor dimers. Proc Natl Acad Sci USA. (2005) 102:9050–5. 10.1073/pnas.0501112102 15932946 PMC1157030

[97] Rashid AJ So CH Kong MM Furtak T El-Ghundi M Cheng R. D1-D2 dopamine receptor heterooligomers with unique pharmacology are coupled to rapid activation of Gq/11 in the striatum. Proc Natl Acad Sci USA. (2007) 104:654–9. 10.1073/pnas.0604049104 17194762 PMC1766439

[98] Mcgraw DW Mihlbachler KA Schwarb MR Rahman FF Small KM Almoosa KF. Airway smooth muscle prostaglandin-EP1 receptors directly modulate beta2-adrenergic receptors within a unique heterodimeric complex. J Clin Invest. (2006) 116:1400–9. 10.1172/JCI25840 16670773 PMC1451203

[99] Tschische P Tadagaki K Kamal M Jockers R Waldhoer M. Heteromerization of human cytomegalovirus encoded chemokine receptors. Biochem Pharmacol. (2011) 82:610–9. 10.1016/j.bcp.2011.06.009 21684267 PMC3156895

[100] Tadagaki K Jockers R Kamal M. History and biological significance of GPCR heteromerization in the neuroendocrine system. Neuroendocrinology. (2012) 95:223–31. 10.1159/000330000 22156565

[101] Tadagaki K Tudor D Gbahou F Tschische P Waldhoer M Bomsel M. Human cytomegalovirus-encoded UL33 and UL78 heteromerize with host CCR5 and CXCR4 impairing their HIV coreceptor activity. Blood. (2012) 119:4908–18. 10.1182/blood-2011-08-372516 22496149

[102] Levoye A Dam J Ayoub MA Guillaume JL Couturier C Delagrange P. The orphan GPR50 receptor specifically inhibits MT1 melatonin receptor function through heterodimerization. EMBO J. (2006) 25:3012–23. 10.1038/sj.emboj.7601193 16778767 PMC1500982

[103] Lohse MJ. Dimerization in GPCR mobility and signaling. Curr Opin Pharmacol. (2010) 10:53–8. 10.1016/j.coph.2009.10.007 19910252

[104] Breit A Lagace M Bouvier M. Hetero-oligomerization between beta2- and beta3-adrenergic receptors generates a beta-adrenergic signaling unit with distinct functional properties. J Biol Chem. (2004) 279:28756–65. 10.1074/jbc.M313310200 15123695

[105] Bouvier M. Oligomerization of G-protein-coupled transmitter receptors. Nat Rev Neurosci. (2001) 2:274–86. 10.1038/35067575 11283750

[106] George SR O'dowd BF Lee SP. G-protein-coupled receptor oligomerization and its potential for drug discovery. Nat Rev Drug Discov. (2002) 1:808–20. 10.1038/nrd913 12360258

[107] Uberti MA Hague C Oller H Minneman KP Hall RA. Heterodimerization with beta2-adrenergic receptors promotes surface expression and functional activity of alpha1D-adrenergic receptors. J Pharmacol Exp Ther. (2005) 313:16–23. 10.1124/jpet.104.079541 15615865

[108] Schelshorn D Joly F Mutel S Hampe C Breton B Mutel V. Lateral allosterism in the glucagon receptor family: glucagon-like peptide 1 induces G-protein-coupled receptor heteromer formation. Mol Pharmacol. (2012) 81:309–18. 10.1124/mol.111.074757 22108912

[109] Gurevich VV Gurevich EV. GPCRs and signal transducers: interaction stoichiometry. Trends Pharmacol Sci. (2018) 39:672–84. 10.1016/j.tips.2018.04.002 29739625 PMC6005764

[110] Lambert NA. GPCR dimers fall apart. Sci Signal. (2010) 3:pe12. 10.1126/scisignal.3115pe12 20354223 PMC3930916

[111] Hern JA Baig AH Mashanov GI Birdsall B Corrie JE Lazareno S. Formation and dissociation of M1 muscarinic receptor dimers seen by total internal reflection fluorescence imaging of single molecules. Proc Natl Acad Sci USA. (2010) 107:2693–8. 10.1073/pnas.0907915107 20133736 PMC2823895

[112] Kasai RS Suzuki KG Prossnitz ER Koyama-Honda I Nakada C Fujiwara TK. Full characterization of GPCR monomer-dimer dynamic equilibrium by single molecule imaging. J Cell Biol. (2011) 192:463–80. 10.1083/jcb.201009128 21300851 PMC3101103

[113] Calebiro D Rieken F Wagner J Sungkaworn T Zabel U Borzi A. Single-molecule analysis of fluorescently labeled G-protein-coupled receptors reveals complexes with distinct dynamics and organization. Proc Natl Acad Sci USA. (2013) 110:743–8. 10.1073/pnas.1205798110 23267088 PMC3545784

[114] Zhu M Wang M Chen Y Zhang C. Pharmacological modulation of two melanocortin-5 receptors by MRAP2 proteins in zebrafish. J Mol Endocrinol. (2018). 10.1530/JME-18-0104. [Epub ahead of print]. 30400043

[115] Schiedel AC Kose M Barreto C Bueschbell B Morra G Sensoy O. Prediction and targeting of interaction interfaces in G-protein coupled receptor oligomers. Curr Top Med Chem. (2018) 18:714–46. 10.2174/1568026618666180604082610 29866008

[116] Wu B Chien EY Mol CD Fenalti G Liu W Katritch V. Structures of the CXCR4 chemokine GPCR with small-molecule and cyclic peptide antagonists. Science. (2010) 330:1066–71. 10.1126/science.1194396 20929726 PMC3074590

[117] Manglik A Kruse AC Kobilka TS Thian FS Mathiesen JM Sunahara RK. Crystal structure of the micro-opioid receptor bound to a morphinan antagonist. Nature. (2012) 485:321–6. 10.1038/nature10954 22437502 PMC3523197

[118] Wu H Wacker D Mileni M Katritch V Han GW Vardy E. Structure of the human kappa-opioid receptor in complex with JDTic. Nature. (2012) 485:327–32. 10.1038/nature10939 22437504 PMC3356457

[119] Park JH Scheerer P Hofmann KP Choe HW Ernst OP. Crystal structure of the ligand-free G-protein-coupled receptor opsin. Nature. (2008) 454:183–7. 10.1038/nature07063 18563085

[120] Scheerer P Park JH Hildebrand PW Kim YJ Krauss N Choe HW. Crystal structure of opsin in its G-protein-interacting conformation. Nature. (2008) 455:497–502. 10.1038/nature07330 18818650

[121] Choe HW Kim YJ Park JH Morizumi T Pai EF Krauss N. Crystal structure of metarhodopsin II. Nature. (2011) 471:651–5. 10.1038/nature09789 21389988

[122] Szczepek M Beyriere F Hofmann KP Elgeti M Kazmin R Rose A. Crystal structure of a common GPCR-binding interface for G protein and arrestin. Nat Commun. (2014) 5:4801. 10.1038/ncomms5801 25205354 PMC4199108

[123] Huang J Chen S Zhang JJ Huang XY. Crystal structure of oligomeric beta1-adrenergic G protein-coupled receptors in ligand-free basal state. Nat Struct Mol Biol. (2013) 20:419–25. 10.1038/nsmb.2504 23435379 PMC3618578

[124] Bakker RA Dees G Carrillo JJ Booth RG Lopez-Gimenez JF Milligan G. Domain swapping in the human histamine H1 receptor. J Pharmacol Exp Ther. (2004) 311:131–8. 10.1124/jpet.104.067041 15159444

[125] Guo W Shi L Filizola M Weinstein H Javitch JA. Crosstalk in G protein-coupled receptors: changes at the transmembrane homodimer interface determine activation. Proc Natl Acad Sci USA. (2005) 102:17495–500. 10.1073/pnas.0508950102 16301531 PMC1287488

[126] Mancia F Assur Z Herman AG Siegel R Hendrickson WA. Ligand sensitivity in dimeric associations of the serotonin 5HT2c receptor. EMBO Rep. (2008) 9:363–9. 10.1038/embor.2008.27 18344975 PMC2271072

[127] Gorinski N Kowalsman N Renner U Wirth A Reinartz MT Seifert R. Computational and experimental analysis of the transmembrane domain 4/5 dimerization interface of the serotonin 5-HT(1A) receptor. Mol Pharmacol. (2012) 82:448–63. 10.1124/mol.112.079137 22669805

[128] George SR Lee SP Varghese G Zeman PR Seeman P Ng GY. A transmembrane domain-derived peptide inhibits D1 dopamine receptor function without affecting receptor oligomerization. J Biol Chem. (1998) 273:30244–8. 10.1074/jbc.273.46.30244 9804783

[129] Yanagawa M Yamashita T Shichida Y. Comparative fluorescence resonance energy transfer analysis of metabotropic glutamate receptors: implications about the dimeric arrangement and rearrangement upon ligand bindings. J Biol Chem. (2011) 286:22971–81. 10.1074/jbc.M110.206870 21550987 PMC3123065

[130] Hu J Hu K Liu T Stern MK Mistry R Challiss RA. Novel structural and functional insights into M3 muscarinic receptor dimer/oligomer formation. J Biol Chem. (2013) 288:34777–90. 10.1074/jbc.M113.503714 24133207 PMC3843091

[131] Sebag JA Hinkle PM. Melanocortin-2 receptor accessory protein MRAP forms antiparallel homodimers. Proc Natl Acad Sci USA. (2007) 104:20244–9. 10.1073/pnas.0708916105 18077336 PMC2154416

[132] Jackson DS Ramachandrappa S Clark AJ Chan LF. Melanocortin receptor accessory proteins in adrenal disease and obesity. Front Neurosci. (2015) 9:213. 10.3389/fnins.2015.00213 26113808 PMC4461818

[133] Yoon YR Lee TG Choi MH Shin SW Ko YG Rhyu IJ. Glucose-regulated protein 78 binds to and regulates the melanocortin-4 receptor. Exp Mol Med. (2018) 50:120. 10.1038/s12276-018-0144-8 30209265 PMC6135830

[134] Rose AS Elgeti M Zachariae U Grubmuller H Hofmann KP Scheerer P. Position of transmembrane helix 6 determines receptor G protein coupling specificity. J Am Chem Soc. (2014) 136:11244–7. 10.1021/ja5055109 25046433

[135] Newman EA Chai BX Zhang W Li JY Ammori JB Mulholland MW. Activation of the melanocortin-4 receptor mobilizes intracellular free calcium in immortalized hypothalamic neurons. J Surg Res. (2006) 132:201–7. 10.1016/j.jss.2006.02.003 16580690

[136] Li XF Lytton J An essential role for the K+-dependent Na+/Ca2+-exchanger, NCKX4, in melanocortin-4-receptor-dependent satiety. J Biol Chem. (2014) 289:25445–59. 10.1074/jbc.M114.564450 25096581 PMC4162149

[137] Buch TR Heling D Damm E Gudermann T Breit A. Pertussis toxin-sensitive signaling of melanocortin-4 receptors in hypothalamic GT1-7 cells defines agouti-related protein as a biased agonist. J Biol Chem. (2009) 284:26411–20. 10.1074/jbc.M109.039339 19648111 PMC2785329

[138] Chai B Li J-Y Zhang W Wang H Mulholland MW. Melanocortin-4 receptor activation inhibits c-Jun N-terminal kinase activity and promotes insulin signaling. Peptides. (2009) 30:1098–104. 10.1016/j.peptides.2009.03.006 19463742 PMC2687409

[139] He S Tao Y-X. Defect in MAPK signaling as a cause for monogenic obesity caused by inactivating mutations in the melanocortin-4 receptor gene. Int J Biol Sci. (2014) 10:1128. 10.7150/ijbs.10359 25332687 PMC4202029

[140] Tao YX. Mutations in melanocortin-4 receptor and human obesity. Progr Mol. Biol. Transl. Sci. (2009) 88:173–204. 10.1016/S1877-1173(09)88006-X 20374728

[141] Hofmann KP Scheerer P Hildebrand PW Choe HW Park JH Heck M. A G protein-coupled receptor at work: the rhodopsin model. Trends Biochem Sci. (2009) 34:540–52. 10.1016/j.tibs.2009.07.005 19836958

[142] Xiang Z Proneth B Dirain ML Litherland SA Haskell-Luevano C. Pharmacological characterization of 30 human melanocortin-4 receptor polymorphisms with the endogenous proopiomelanocortin-derived agonists, synthetic agonists, and the endogenous agouti-related protein antagonist. Biochemistry. (2010) 49:4583–600. 10.1021/bi100068u 20462274 PMC2888279

[143] Melchior C Schulz A Windholz J Kiess W Schöneberg T Körner A. Clinical and functional relevance of melanocortin-4 receptor variants in obese German children. Horm Res Paediatr. (2012) 78:237–46. 10.1159/000343816 23146882

[144] Moore BS Mirshahi UL Yost EA Stepanchick AN Bedrin MD Styer AM. Long-term weight-loss in gastric bypass patients carrying melanocortin 4 receptor variants. PLoS ONE. (2014) 9:e93629. 10.1371/journal.pone.0093629 24705671 PMC3976318

[145] Zhang Y Sun B Feng D Hu H Chu M Qu Q. Cryo-EM structure of the activated GLP-1 receptor in complex with a G protein. Nature. (2017) 546:248–53. 10.1038/nature22394 28538729 PMC5587415

[146] Sounier R Mas C Steyaert J Laeremans T Manglik A Huang W. Propagation of conformational changes during mu-opioid receptor activation. Nature. (2015) 524:375–8. 10.1038/nature14680 26245377 PMC4820006

[147] Carpenter B Nehmé R Warne T Leslie AG Tate CG Structure of the adenosine A2A receptor bound to an engineered G protein. Nature. (2016) 536:104–07. 10.1038/nature18966 27462812 PMC4979997

[148] Zhang J Li X Zhou Y Cui L Li J Wu C. The interaction of MC3R and MC4R with MRAP2, ACTH, alpha-MSH and AgRP in chickens. J Endocrinol. (2017) 234:155–74. 10.1530/JOE-17-0131 28512117

[149] Hinney A Bettecken T Tarnow P Brumm H Reichwald K Lichtner P. Prevalence, spectrum, and functional characterization of melanocortin-4 receptor gene mutations in a representative population-based sample and obese adults from Germany. J Clin Endocrinol Metab. (2006) 91:1761–9. 10.1210/jc.2005-2056 16492696

