# Supplementary material for: Signal Transduction and Pathogenic Modifications at the Melanocortin-4 Receptor: A Structural Perspective
Source: Front Endocrinol (Lausanne). 2019 Jul 31;10:515. doi: 10.3389/fendo.2019.00515 (PMC6685040; doi:10.3389/fendo.2019.00515)
Supplement: Supplementary file 1 [file Data_Sheet_1.PDF]

SUPPLEMENTARY MATERIAL

Supplementary table 1

Receptor region	Wild type residue	B & W numbering	Pathogenic Substitution	Functional characterization (MSH binding or cAMP signaling)	References
Ntt	*S4*		S4F	=	(19,20)
	H6		H6P	no data	(21)
	R7		R7C	= (1,2), -- (3)	(1-3,22)
	R7		R7H	= (4-6)	(4-6,23,24)
	T11		T11S	=	(5,9,13,25)
	T11		T11A	= (6-10), -- (8,11)	(6-9,11,15,16,26)
	R18		R18C	= (9,12-14), -- (3)	(1,3,9,12-15)
	R18		R18H	= (5,13-15), -- (16)	(5,13-16)
	R18		R18L	=	(4,5,13)
	L23		L23R	no data	(27)
	S30		S30F	=	(9,19,24,28-32)
	G32		G32E	=	(19,33)
	G34		G34A	=	(20)
	Y35		Y35C	=	(34)
	S36		S36Y	=	(4,35)
	S36		S36T	no data	(10,36,37)
	D37		D37V	- (16)	(15,16,29)
	D37		D37G	= (17)	(17)
	C40		C40R	=	(34,38)
	C40		C40Y	= (17,18)	(17,18)
	E42		E42K	no data	(39)
TMH1	*P48*	1.36	P48S	= (4,24,40)	(4,40,47,48)
	V50	1.38	V50M	=	(4,14,24,40,49)
	F51	1.39	F51L	= (8,26), - (4)	(4,8,15,22,26)
	L54	1.42	L54P	-	(41)
	G55	1.43	G55V*	-- (41-43)	(41-43,50)
	G55	1.43	G55D*	--	(41)
	S58	1.46	S58C	--	(14,16,24,40,49,51,52)
	S58	1.46	S58N	no data	(21)
	E61	1.49	E61K	--	(4,19,33,41,51)
	N62	1.50	N62S	- (16,44), -- (4,11,19,37,45,46)	(11,16,18,24,37,40,44-46,51,52)
	V65	1.53	V65E	no data	(53)
	I69	1.57	I69M*	---	(13,15)
	I69	1.57	I69R*	---	(46,52,54,55)
	I69	1.57	I69T*	--	(4,51,56)
	A70	1.58	A70T	=	(1)
ICL1	*N72*		N72K	-	(57)

	K73		K73R	no data	(58)
	N74		N74I	=	(59)
	H76		H76R	- (15,20)	(15,20,55)
	S77		S77L	=	(60)
**TMH2**	***P78***	2.38	P78L	-- (61), --- (16,24,62)	(9,24,29,40,46,52,61,62,72)
	M79	2.39	M79I	- (55)	(54,55)
	C84	2.44	C84R	--	(2,22,46,52)
	A87	2.47	A87D	---	(73)
	D90	2.50	D90N	--- (4,20,63)	(4,20,63,70)
	S94	2.54	S94R	---	(4,61)
	S94	2.54	S94N	--	(15,55)
	V95	2.55	V95I	- (64) --- (16,61)	(15,16,61)
	N97	2.56	N97D*	-- (45,65), --- (11,16,66)	(11,13,16,20,45,65,66)
	G98	2.57	G98R*	---	(4,52,65,74,75)
	T101	2.58	T101A	--	(1)
	T101	2.58	T101N	no data	(76)
	I102	2.62	I102S	-- (16), --- (14,24)	(8,14,16,24,49,65)
	I102	2.62	I102T	= (14,35), - (13)	(8,9,13-15,24,35,77)
	V103	2.63	V103I	= (16,28,61,67-71)	(6,12,15,16,19,20,22,29,48-50,61,67-71,77-79)
	L106	2.66	L106P	- (65) --- (11,45)	(11,16,45,65)
**ECL1**	***T112***		T112K	---	(1)
	T112		T112M	= (9,19,61,62,70,78), - (8,67)	(1,8,15,16,19,20,26,29,48,61,62,67,70,77,78,80)
**TMH3**	***I121***	3.24	I121T	= (4) -- (61)	(4,61)
	N123	3.26	N123S	no data	(82)
	I125	3.28	I125K*	-- (45), --- (11,16)	(11,16,45)
	D126	3.29	D126Y	---	(15,55)
	S127	3.30	S127L	-- (14,16,24,28,33,71)	(2,6,14,15,19,22,24,30,32,48,61,65,66,71,78,83)
	V128	3.31	V128L	--	(10)
	S136	3.39	S136F	--	(84)
	S136	3.39	S136A	no data	(82)
	S136	3.39	S136P	--- (56,81)	(2,56,81,84)
	I137	3.40	I137T	= (62), - (16,67)	(6,16,20,62,67)
	A144	3.47	A144L	no data	(85)
	D146	3.49	D146H	no data	(86)
	D146	3.49	D146N	= (15,55)	(15,55,86)
	R147	3.50	R147G	no data	(23)
	T150	3.53	T150F	--	(12)
	T150	3.53	T150I	-- (16,62), --- (13)	(13,15,16,62)
**ICL2**	***A154****		A154D	= (4,8,66), -- (13)	(4,8,13,15,66)
	Q156		Q156R	=	(1)
	Q156		Q156P	=	(6,66)
	Y157		Y157S	-- (4,87)	(4,40,46,52,87)

	H158		H158R	=, CAM	(1,19)
	M161		M161T	-- (41)	(41,88)
	T162		T162R	=	(89)
	T162		T162I	= (41)	(41,51,90)
**TMH4**	***R165***	4.41	R165G	= (43) -- (69)	(43,69)
	R165	4.41	R165W*	-- (9,13,25,61,62,70)	(1,7,9,12,13,15,24,51,61,62,70,72)
	R165	4.41	R165Q*	-- (1,11,45,62,91), --- (35)	(1,11,24,35,43-45,51,62,69,92,93)
	V166	4.42	V166I	= (1,37,71)	(1,18,27,37,71)
	I169	4.45	I169S	= (16,19)	(16)
	I170	4.46	I170V	= (9,13,37,62) - (13,25)	(9,13,15,24,25,37,40,49,62)
	C172	4.48	C172R	--- (43,50)	(43,50)
	W174	4.50	W174C*	--- (4)	(2,4,46,52,80,94)
	A175	4.51	A175T *	= (16) (60), -- (11,12,45)	(6,11,12,16,19,45,80,94) (60)
	T178	4.54	T178M	=	(16,61)
	V179	4.55	V179A	no data	(95)
	G181	4.57	G181D	--- (4,35,61)	(1,4,22,35,61,70)
**ECL2**	***I185***	4.61	I185F	--- (10)	(10)
	I186	4.62	I186V	= (55)	(55,96)
**TMH5**	***S191***		S191T	=	(17)
	I194		I194T*	---	(55)
	I195		I195S	-	(54,55)
	I195		I195V	=	(19)
	M200	5.36	M200V	= (1,62), - (8)	(1,8,15,26,62)
	F201	5.37	F201L	= (55), -- (15)	(15,55)
	F202	5.38	F202L	= (4,19,37,43,77)	(4,6,8,15,19,20,37,43,50,77)
	L207	5.43	L207V	=	(20,83)
	M208	5.44	M208V	-- (43)	(43,50)
	M215	5.51	M215L	no data	(58)
	M218	5.54	M218T	no data	(34)
	A219	5.55	A219V*	= (35) -- (4)	(2,4,35)
**ICL3**	***I226***		I226T	- (4,78)	(1,4,78)
	P230		P230L	= (16), constitutively active (2,61)	(2,16,61)
	G231		G231S	= (4,13,14,19,66)	(4,13-15,18,19,66,69,92)
	G231		G231V	=	(15,55)
	R236		R236C	=	(19,98)
	G238		G238D	= (4) - (97)	(4,11,97)
	A239		A239V	no data	(21)
**TMH6**	***N240***	6.30	N240S	= (4,8,19)	(4,6,8,19,77)
	A244	6.34	A244E	- (16,24), -- (61)	(15,16,24,61)
	A244	6.34	A244V	= (99)	(99)
	T246	6.36	T246A	-	(10)
	T248	6.48	T248A	=	(75)
	L250	6.40	L250Q	= (13,25,62), - (16)	(12,13,15,16,24,62)

	I251	6.41	I251L	= (12,69-71), - (16)	(6,12,16,20,22,25,28,29,49,50,61,69-71,78,79)
	I251	6.41	I251F	- (10)	(10)
	G252	6.42	G252S	- (1,16,29,61,100)	(1,6,14-16,61,100)
	V253	6.43	V253I	= (16,24,45,62) - (11,64)	(1,6,11,15,16,20,24,44,45,62,66,79)
	P260	6.50	P260Q*	-- (15,96), --- (55)	(15,46,52,55,96)
	F261	6.51	F261S*	-- [100]	(2,46,52,101)
	L263	6.53	L263V	no data	(6)
	H264	6.54	H264R	no data	(21)
**ECL3**	***I269***		I269N*	- (19,43,69) -- (56)	(19,43,50,56,69)
	C271		C271Y*	-- (16,38), --- (9,11,45,102)	(7,9,16,24,38,40,44-46,51,52)
	C271		C271R	= (11,62) - (38)	(4,11,15,38,62)
	C271		C271F	-	(19,98)
	P272		P272L	= (72), -- (59)	(59,72)
	N274		N274S	= (11,16,77)	(11,15,16,77,103)
	P275		P275S	= (69)	(30,43,69)
	F280		F280L	--	(55,96)
	M281		M281V	-	(99)
**TMH7**	***I289***	7.40	I289L	---	(15,55)
	C293	7.44	C293R	no data	(105)
	S295	7.46	S295P	= (8,13)	(4,8,13,15,20)
	P299	7.50	P299H	--- (14,16,19,24,33)	(6,14-16,19,24,33,51,66)
	P299	7.50	P299S	--- (19)	(19,98)
	P299	7.50	P299L	--- (4)	(4)
	L300	7.51	L300Q	no data	(21)
	I301	7.52	I301T	- (12,13,62), -- (16)	(12,13,15,16,62)
	Y302	7.53	Y302F	no data	(106)
	A303	7.54	A303T	-- (33,41)	(19,33,41)
	A303	7.54	A303P*	-- (104)	(104,107)
	L304	7.55	L304F	=	(18,69)
	R305	7.56	R305W	---	(13,14)
	R305	7.56	R305S	- (55), -- (15)	(15,55)
	R305	7.56	R305Q	- (19)	(19,20,48)
**H8**	***E308***	8.49	E308K	-	(4,108)
	R310	8.51	R310K	no data	(27)
	I316	8.57	I316S	-- (11,16,45)	(11,16,45,72,88)
	I317	8.58	I317T	= (16,24,61,62)	(16,24,29,61,62,109)
	I317	8.58	I317V	=	(4,80,110)
**Cterm**	***G323***		G323E	no data	(23)
	L325		L325F	---	(35)
	C326		C326R	=	(33)
	R331		R331K	=	(6)
	Y332		Y332C	=	(15,55)
	Y332		Y332H	=	(15,55)

Supplementary table 1: Pathogenic MC4R single side-chain substitutions.

To systematically review available literature on missense singe amino acid substitutions we searched (time point June 2017) all publications regarding the term “MC4R”, or “mc4-r and mutations”, or “pathogenic mutation”, or “MC4R and substitution” in Google Scholar (7588 hits). Only peer-reviewed data of missense mutations were taken into consideration and stop mutations, deletions and double substitutions were excluded. Pathogenic single side chain substitutions reported for MC4R are listed based on their primary sequence position, the structural receptor region and the unifying Ballesteros & Weinstein numbering system (111). Functional characterizations were implemented in a simplified sorting system (either MSH binding affinity and/or cAMP signaling) for each experimental report as follows: substitutions resulting in a decreased functional range less than two-fold compared to wt were classified as like wt (“=”, gray background), for 2-10 fold decreased functional parameters “-” was used, down to ten-fold data were indicated “--”, and a total loss-of-signaling is indicated as “---”. Furthermore, constitutively active and functionally uncharacterized substitutions are indicated (no data available). Single side chain substitutions reported yet to be linked to biased signaling are highlighted by an asterix *. Multiple identification and investigation of specific substitutions are indicated by respective citations of the corresponding literature.

Supplementary table 2

structural region	A) number of amino acids in the structural part	B) substituted amino acid positions (total number of variations)	B/A ratio [%]	no functional data available	like wild type (in cAMP accumulation or MSH binding)	decreased signaling (cAMP accumulation or MSH binding)
**N-term**	45	14 (21)	31	4	16 (11)	5 (11)
**TMH1**	25	11 (15)	44	2	4 [1]	10 [1]
**ICL1**	8	5 (5)	50	1	2	2
**TMH2**	30	13 (16)	43	1	2 [1]	14 [1]
**ECL1**	12	1 (2)	8	-	1 [1]	2 [1]
**TMH3**	30	12 (16)	40	5	3 [2]	10 [2]
**ICL2**	13	6 (8)	43	-	6 [1]	3 [1]
**TMH4**	21	10 (12)	48	1	6 [3]	8 [3]
**ECL2**	7	2 (2)	29	-	1	1
**TMH5**	30	11 (12)	37	2	7 [3]	6 [3]
**ICL3**	18	6 (7)	33	1	5 [1]	2 [1]
**TMH6**	30	12 (14)	40	2	6 [3]	9 [3]
**ECL3**	16	7 (9)	44	0	4 [2]	7 [2]
**TMH7**	21	10 (15)	47	3	2	10
**H8**	9	4 (5)	44	1	2	2
**Cterm**	13	5 (6)	29	1	4	1
**Total**	**332**	**129 (165)**	**39**	**24**	**71 [22]**	**92 [22]**

Supplementary table 2: Prevalence, locations and functional information on pathogenic MC4R single amino acid substitution.
The table shows statistical evaluation of information concerning pathogenic mutations at the MC4R (Supplementary table 1). The ratio (in %) of the number of residues in a specific structural receptor region (A) and the number of single substituted amino acids (B) reflects the frequency of mutated positions in a certain receptor region and might be a hint for a specific functional importance of these parts. A so far known functional impact of each mutation is reflected additionally in the right three columns (remark: Several mutations are reported in different studies controversially as like wt or as decreased in signaling, respectively). The length of specific structural receptor regions was determined according to the provided homology model (Figure 2).

Supplementary Figure 1

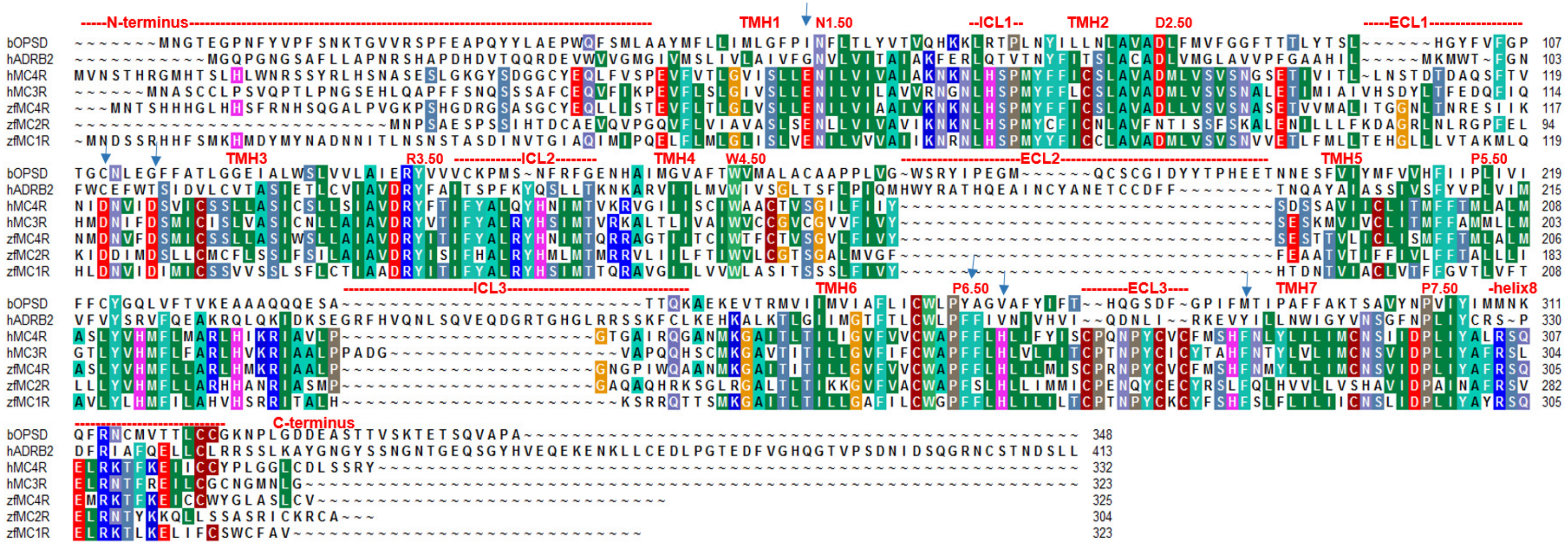

Supplementary figure 1: Amino acid sequence comparison between different human and zebra fish MCRs and prototypical GPCRs. This manually adjusted sequence alignment includes bovine rhodopsin (bOPSD) and the β-2 adrenergic receptor (ADRB2) in comparison to human MC4R, MC3R and several zfMCRs. This comparison is helpful to define structural dimensions of each receptor part, like the helices or loops. These dimensions are indicated above the sequences (ICL = intracellular loops, ECLs = extracellular loops, TMH = transmembrane helices). Highly conserved positions according to the unifying *Ballesteros and Weinstein* numbering scheme for class A GPCRs (112) are indicated by respective numbers. Moreover, arrows indicating conserved amino acids among MCRs involved in MSH binding. This alignment was visualized with the software BioEdit. Specific background colors indicating conservation (Blossum62 matrix) and reflecting biophysical properties of the amino acid side chains: black – proline, blue – positively charged, cyan/green – aromatic and hydrophobic, green – hydrophobic, red – negatively charged, gray – hydrophilic, dark-red – cysteines, magenta – histidine.

Supplementary Figure 2

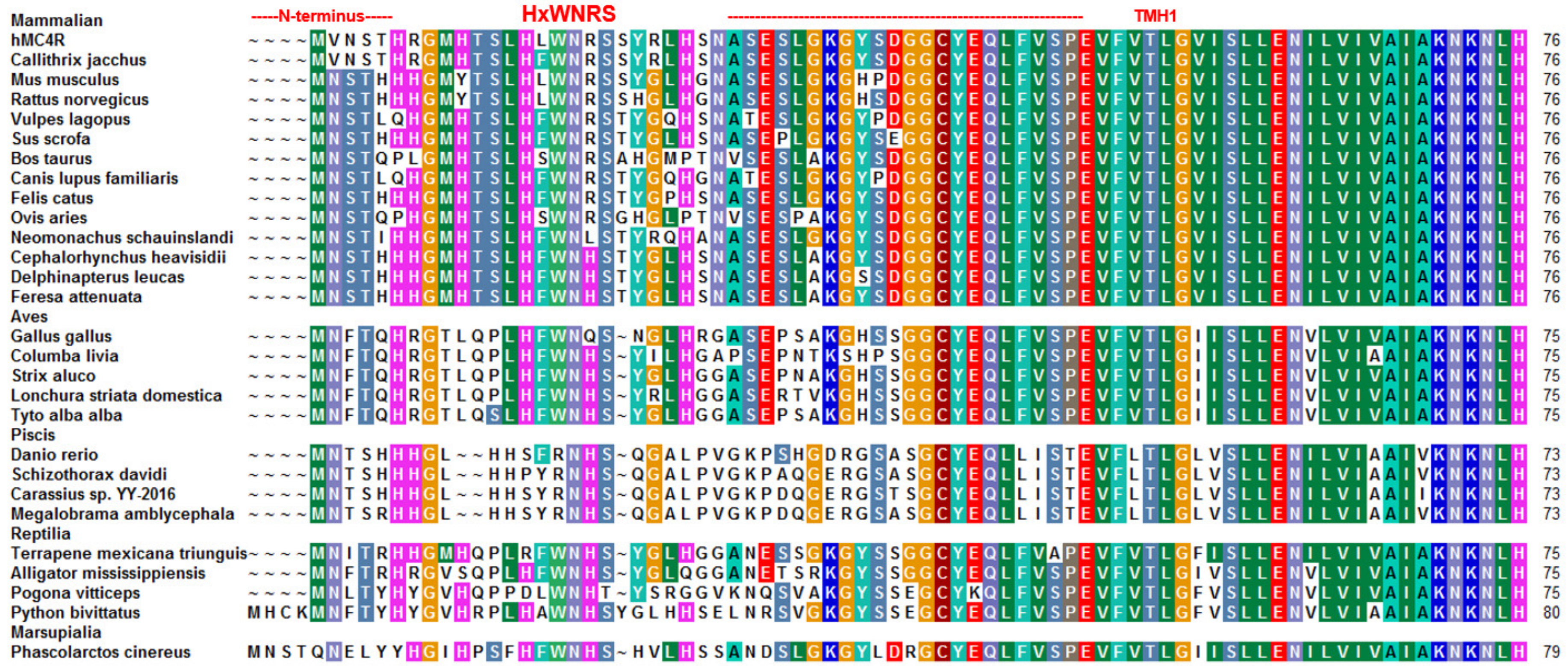

Supplementary figure 2: Sequence comparison of the N-terminus of MC4R orthologous.
The sequence alignment of different MC4R species (e.g. birds, fish's, different mammals) visualizes conservation of amino acids at corresponding positions in the N-terminus. Of note, the HxWNRS motif is discussed to have a fundamental role in hMC4R for signaling regulation (113). Specific background colors indicating conservation (Blossum62 matrix) and reflecting biophysical properties of the amino acid side chains: black – proline, blue – positively charged, cyan/green – aromatic and hydrophobic, green – hydrophobic, red – negatively charged, gray – hydrophilic, dark-red – cysteine, magenta – histidine.

Supplementary Figure 3

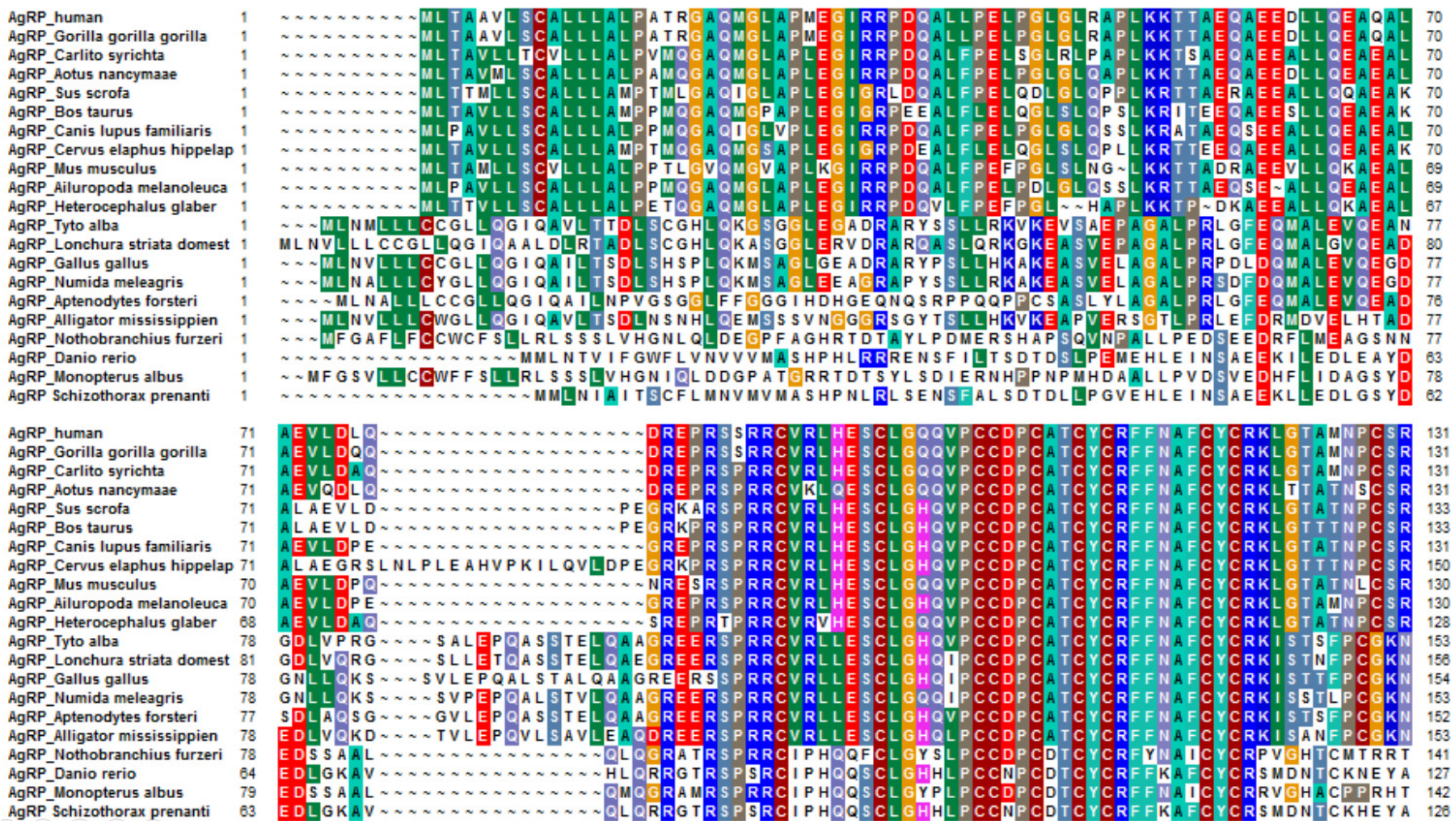

Supplementary figure 3: Sequence comparison between AgRP orthologous.
The sequence alignment (manually adjusted) of different species (e.g. birds, fish's, different mammals) visualizes conservation of amino acids at

corresponding AgRP peptide positions. Of note, while the C-terminal part is highly conserved among all species (between position ~80 and the C-terminus) and is characterized by several cysteines and respective disulfide bridges (see Figure 4), the N-terminus is not highly conserved among different animal classes and not stabilized by disulfide bridges. Specific background colors indicating conservation (Blossum62 matrix) and reflecting biophysical properties of the amino acid side chains: black – proline, blue – positively charged, cyan/green – aromatic and hydrophobic, green – hydrophobic, red – negatively charged, gray – hydrophilic, dark-red – cysteine, magenta – histidine.

Supplementary Figure 4

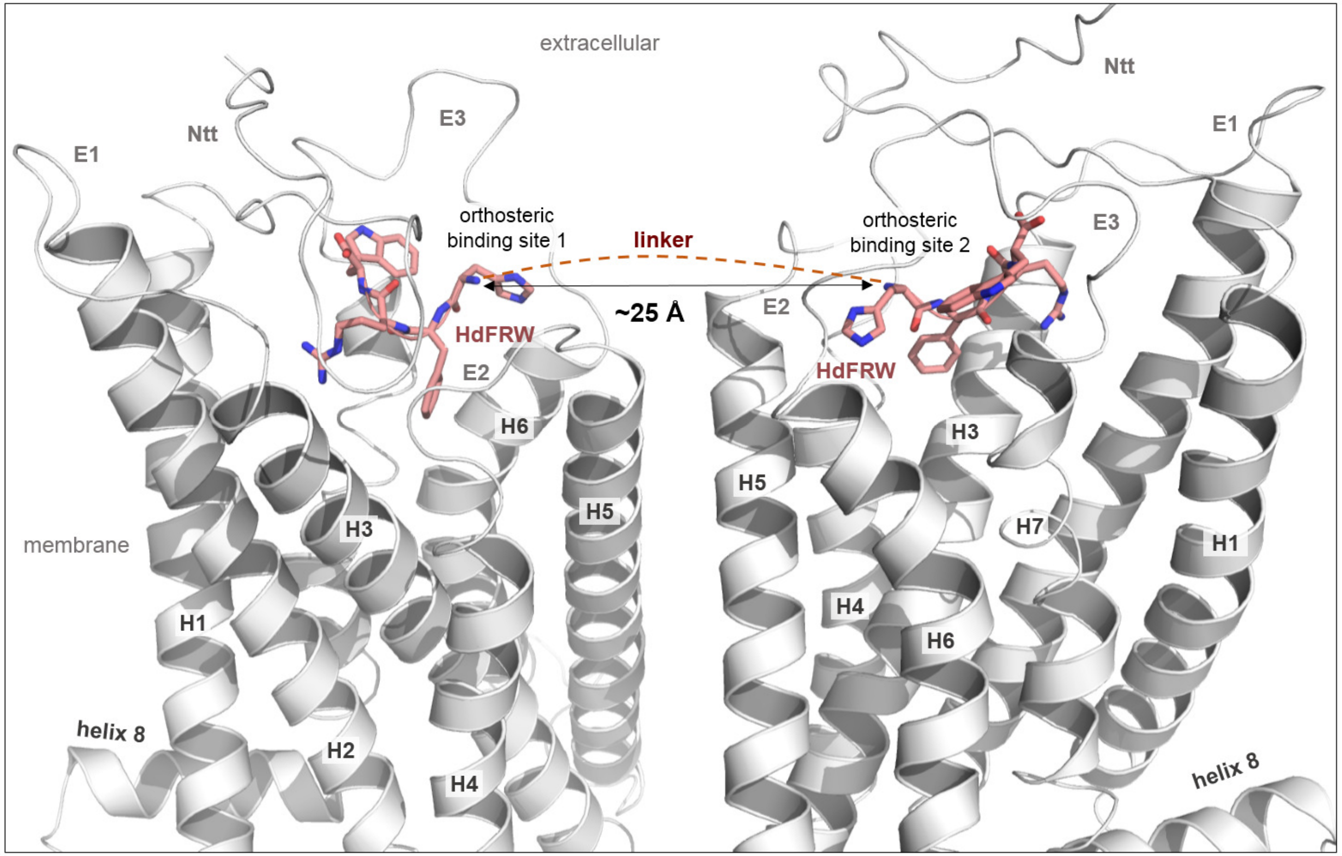

Supplementary figure 4: MC4R homodimer model with implications for binding of peptidic bivalent agonistic ligands.
Recently a homobivalent agonist (CJL-1-87) with a 20 atoms spanning linker connecting two HdFRW moieties (core amino acids of MSH, stick representation in light red) was reported (114,115). It was suggested that the unique pharmacology of this ligand may be a result either of interactions with MC3R homodimers, MC4R homodimers, or heterodimers between MC3R and MC4R. Here we show exemplarily that in a putative MC4R homodimer constellation with a TMH4/5 - TMH4/5 interface (Figure 7A) the two moieties can be located in the suggested orthosteric (MSH) binding site (see Figure 3), whereby a distance of approximately 25 Å between the ligands would be ideal for this specific linker (oriented above the E2).

Supplementary Figure 5

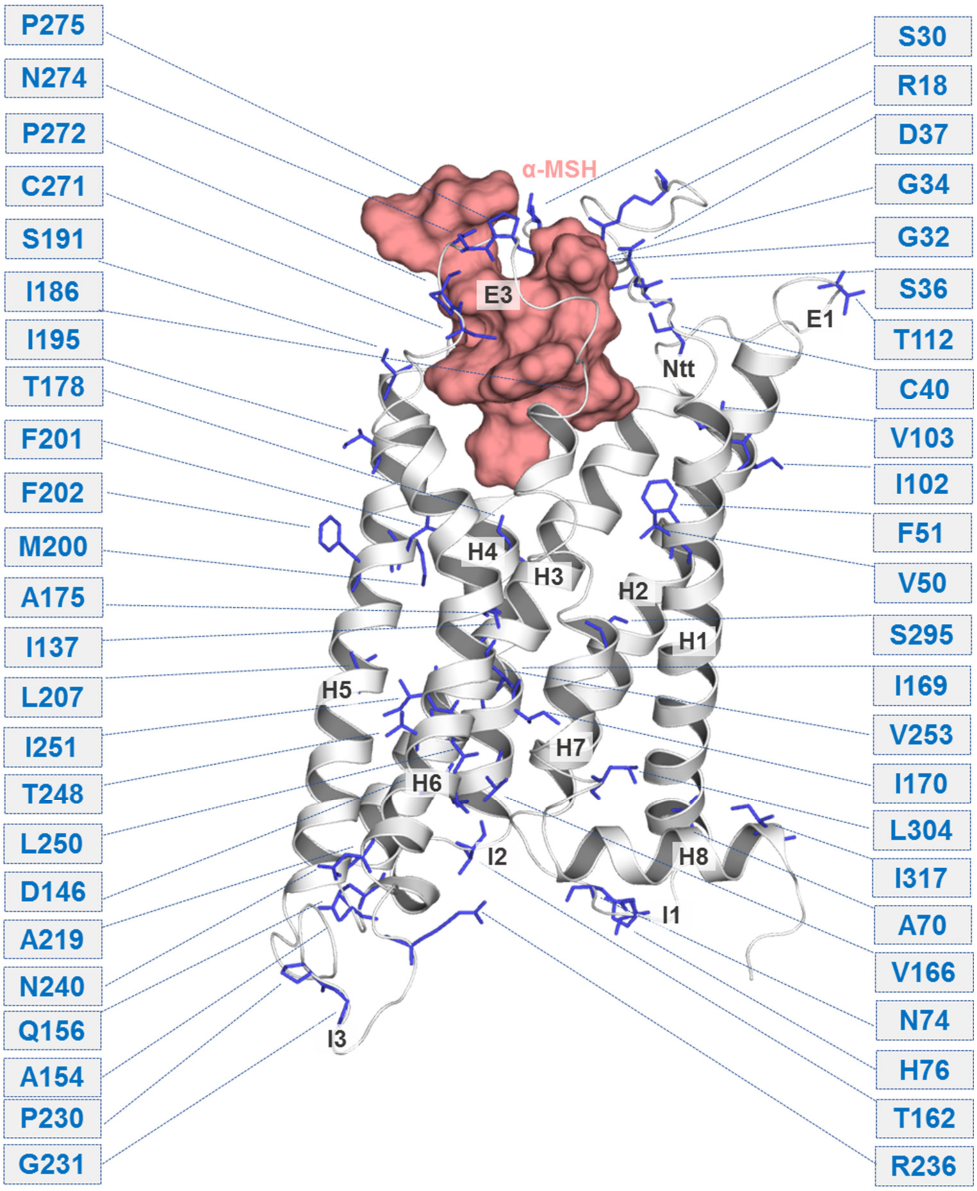

Supplementary figure 5: Wild-type positions of naturally occurring MC4R mutations that are yet characterized as "like wild type" in cell-based assays (Gs mediated signaling). Several MC4R mutations were identified in obese patients that are reported as functionally comparable to wild type (Supplementary table 1). This comparison is based on cell-based assays for MSH binding or cAMP production. These wild type amino acids (blue sticks, 48 positions are highlighted) carry different 55 mutations (7 positions are not part of the model at the Ntt or Ctt) and they are highlighted at the 3D-model of the MC4R/α-MSH complex (cartoon and surface representation).

References

1. Hinney, A., Bettecken, T., Tarnow, P., Brumm, H., et al. (2006) Prevalence, spectrum, and functional characterization of melanocortin-4 receptor gene mutations in a representative population-based sample and obese adults from Germany. *The Journal of Clinical Endocrinology & Metabolism* 91, 1761-1769
2. Fan, Z. C., and Tao, Y. X. (2009) Functional characterization and pharmacological rescue of melanocortin-4 receptor mutations identified from obese patients. *Journal of cellular and molecular medicine* 13, 3268-3282
3. Srinivasan, S., Lubrano-Berthelier, C., Govaerts, C., Picard, F., et al. (2004) Constitutive activity of the melanocortin-4 receptor is maintained by its N-terminal domain and plays a role in energy homeostasis in humans. *The Journal of clinical investigation* 114, 1158-1164
4. Xiang, Z., Proneth, B., Dirain, M. L., Litherland, S. A., and Haskell-Luevano, C. (2010) Pharmacological characterization of 30 human melanocortin-4 receptor polymorphisms with the endogenous proopiomelanocortin-derived agonists, synthetic agonists, and the endogenous agouti-related protein antagonist. *Biochemistry* 49, 4583-4600
5. Srinivasan, S., Lubrano-Berthelier, C., Govaerts, C., Picard, F., et al. (2004) Constitutive activity of the melanocortin-4 receptor is maintained by its N-terminal domain and plays a role in energy homeostasis in humans. *J Clin Invest* 114, 1158-1164
6. Hatoum, I. J., Stylopoulos, N., Vanhoose, A. M., Boyd, K. L., et al. (2012) Melanocortin-4 receptor signaling is required for weight loss after gastric bypass surgery. *The Journal of Clinical Endocrinology & Metabolism* 97, E1023-E1031
7. Farooqi, I. S., Yeo, G. S., and O'Rahilly, S. (2003) Binge eating as a phenotype of melanocortin 4 receptor gene mutations. *The New England journal of medicine* 349, 606-609; author reply 606-609
8. Tao, Y.-X., and Segaloff, D. L. (2005) Functional analyses of melanocortin-4 receptor mutations identified from patients with binge eating disorder and nonobese or obese subjects. *The Journal of Clinical Endocrinology & Metabolism* 90, 5632-5638
9. Xiang, Z., Litherland, S. A., Sorensen, N. B., Proneth, B., et al. (2006) Pharmacological characterization of 40 human melanocortin-4 receptor polymorphisms with the endogenous proopiomelanocortin-derived agonists and the agouti-related protein (AGRP) antagonist. *Biochemistry* 45, 7277-7288
10. Bonnefond, A., Keller, R., Meyre, D., Stutzmann, F., et al. (2016) Eating Behavior, Low-Frequency Functional Mutations in the Melanocortin-4 Receptor (MC4R) Gene, and Outcomes of Bariatric Operations: A 6-Year Prospective Study. *Diabetes care* 39, 1384-1392
11. Farooqi, I. S., Keogh, J. M., Yeo, G. S., Lank, E. J., et al. (2003) Clinical spectrum of obesity and mutations in the melanocortin 4 receptor gene. *New England Journal of Medicine* 348, 1085-1095
12. Vaisse, C., Clement, K., Durand, E., Hercberg, S., et al. (2000) Melanocortin-4 receptor mutations are a frequent and heterogeneous cause of morbid obesity. *The Journal of clinical investigation* 106, 253-262
13. Lubrano-Berthelier, C., Dubern, B., Lacorte, J.-M., Picard, F., et al. (2006) Melanocortin 4 receptor mutations in a large cohort of severely obese adults: prevalence, functional classification, genotype-phenotype relationship, and lack of association with binge eating. *The Journal of Clinical Endocrinology & Metabolism* 91, 1811-1818
14. Roubert, P., Dubern, B., Plas, P., Lubrano-Berthelier, C., et al. (2010) Novel pharmacological MC4R agonists can efficiently activate mutated MC4R from obese patient with impaired endogenous agonist response. *Journal of endocrinology* 207, 177-183
15. Stutzmann, F., Tan, K., Vatin, V., Dina, C., et al. (2008) Prevalence of melanocortin-4 receptor deficiency in Europeans and their age-dependent penetrance in multigenerational pedigrees. *Diabetes* 57, 2511-2518
16. Xiang, Z., Litherland, S. A., Sorensen, N. B., Proneth, B., et al. (2006) Pharmacological Characterization of 40 Human Melanocortin-4 Receptor Polymorphisms with the Endogenous Proopiomelanocortin-Derived Agonists and the Agouti-Related Protein (AGRP) Antagonist†. *Biochemistry* 45, 7277-7288

17. Hughes, D. A., Hinney, A., Brumm, H., Wermter, A.-K., et al. (2009) Increased constraints on MC4R during primate and human evolution. *Human genetics* 124, 633
18. Berg, L., Beekum, O., Heutink, P., Felius, B. A., et al. (2011) Melanocortin-4 Receptor Gene Mutations in a Dutch Cohort of Obese Children. *Obesity* 19, 604-611
19. Calton, M. A., Ersoy, B. A., Zhang, S., Kane, J. P., et al. (2009) Association of functionally significant Melanocortin-4 but not Melanocortin-3 receptor mutations with severe adult obesity in a large North American case-control study. *Hum Mol Genet* 18, 1140-1147
20. Moore, B. S., Mirshahi, U. L., Yost, E. A., Stepanchick, A. N., et al. (2014) Long-term weight-loss in gastric bypass patients carrying melanocortin 4 receptor variants. *PLoS One* 9, e93629
21. Kirac, D., Kasimay Cakir, O., Avcilar, T., Deyneli, O., et al. (2016) Effects of MC4R, FTO, and NMB gene variants to obesity, physical activity, and eating behavior phenotypes. *IUBMB life* 68, 806-816
22. Hainerová, I., Larsen, L. H., Holst, B., Finková, M., et al. (2007) Melanocortin 4 receptor mutations in obese Czech children: studies of prevalence, phenotype development, weight reduction response, and functional analysis. *The Journal of Clinical Endocrinology & Metabolism* 92, 3689-3696
23. Albuquerque, D., Estévez, M. N., Víbora, P. B., Giralt, P. S., et al. (2014) Novel variants in the MC4R and LEPR genes among severely obese children from the Iberian population. *Annals of human genetics* 78, 195-207
24. Lubrano-Berthelier, C., Durand, E., Dubern, B., Shapiro, A., et al. (2003) Intracellular retention is a common characteristic of childhood obesity-associated MC4R mutations. *Human molecular genetics* 12, 145-153
25. Vaisse, C., Clement, K., Durand, E., Hercberg, S., et al. (2000) Melanocortin-4 receptor mutations are a frequent and heterogeneous cause of morbid obesity. *J Clin Invest* 106, 253-262
26. Branson, R., Potoczna, N., Kral, J. G., Lentes, K.-U., et al. (2003) Binge eating as a major phenotype of melanocortin 4 receptor gene mutations. *New England Journal of Medicine* 348, 1096-1103
27. Wang, C., Liang, L., Wang, H., Fu, J. F., et al. (2006) Several mutations in the melanocortin 4 receptor gene are associated with obesity in Chinese children and adolescents. *Journal of endocrinological investigation* 29, 894-898
28. Hinney, A., Hohmann, S., Geller, F., Vogel, C., et al. (2003) Melanocortin-4 receptor gene: case-control study and transmission disequilibrium test confirm that functionally relevant mutations are compatible with a major gene effect for extreme obesity. *J Clin Endocrinol Metab* 88, 4258-4267
29. Hinney, A., Schmidt, A., Nottebom, K., Heibult, O., et al. (1999) Several mutations in the melanocortin-4 receptor gene including a nonsense and a frameshift mutation associated with dominantly inherited obesity in humans. *The Journal of Clinical Endocrinology & Metabolism* 84, 1483-1486
30. Hinney, A., Bettecken, T., Tarnow, P., Brumm, H., et al. (2006) Prevalence, spectrum, and functional characterization of melanocortin-4 receptor gene mutations in a representative population-based sample and obese adults from Germany. *J Clin Endocrinol Metab* 91, 1761-1769
31. Hinney, A., Schmidt, A., Nottebom, K., Heibult, O., et al. (1999) Several mutations in the melanocortin-4 receptor gene including a nonsense and a frameshift mutation associated with dominantly inherited obesity in humans. *J Clin Endocrinol Metab* 84, 1483-1486
32. Stutzmann, F., Tan, K., Vatin, V., Dina, C., et al. (2008) Prevalence of melanocortin-4 receptor deficiency in Europeans and their age-dependent penetrance in multigenerational pedigrees. *Diabetes* 57, 2511-2518
33. Ahituv, N., Kavaslar, N., Schackwitz, W., Ustaszewska, A., et al. (2007) Medical sequencing at the extremes of human body mass. *The American Journal of Human Genetics* 80, 779-791
34. Rong, R., Tao, Y. X., Cheung, B. M., Xu, A., et al. (2006) Identification and functional characterization of three novel human melanocortin-4 receptor gene variants in an obese Chinese population. *Clinical endocrinology* 65, 198-205

35. Larsen, L. H., Echwald, S. M., Sørensen, T. I., Andersen, T., et al. (2005) Prevalence of mutations and functional analyses of melanocortin 4 receptor variants identified among 750 men with juvenile-onset obesity. *The Journal of Clinical Endocrinology & Metabolism* 90, 219-224
36. Logan, M., der Merwe, V., Dodgen, T. M., Myburgh, R., et al. (2016) Allelic variants of the Melanocortin 4 receptor (MC4R) gene in a South African study group. *Molecular genetics & genomic medicine* 4, 68-76
37. Hughes, D. A., Hinney, A., Brumm, H., Wermter, A. K., et al. (2009) Increased constraints on MC4R during primate and human evolution. *Hum Genet* 124, 633-647
38. Tarnow, P., Schöneberg, T., Krude, H., Grüters, A., and Biebermann, H. (2003) Mutationally induced disulfide bond formation within the third extracellular loop causes melanocortin 4 receptor inactivation in patients with obesity. *Journal of Biological Chemistry* 278, 48666-48673
39. Dar, R., Rasool, S., Zargar, A. H., Jan, T. R., and Andrabi, K. I. (2015) Association of MC4R codon 42 polymorphism with obese/diabetic ethnic Kashmiri population. *Asian Journal of Medical and Biological Research* 1, 149-157
40. Tao, Y.-X., and Segaloff, D. L. (2003) Functional characterization of melanocortin-4 receptor mutations associated with childhood obesity. *Endocrinology* 144, 4544-4551
41. Tan, K., Pogozheva, I. D., Yeo, G. S., Hadaschik, D., et al. (2009) Functional characterization and structural modeling of obesity associated mutations in the melanocortin 4 receptor. *Endocrinology* 150, 114-125
42. Cai, G., Cole, S. A., Butte, N., Bacino, C., et al. (2006) A quantitative trait locus on chromosome 18q for physical activity and dietary intake in Hispanic children. *Obesity* 14, 1596-1604
43. Hohenadel, M. G., Thearle, M. S., Grice, B. A., Huang, H., et al. (2014) Brain-derived neurotrophic factor in human subjects with function-altering melanocortin-4 receptor variants. *International journal of obesity (2005)* 38, 1068
44. Farooqi, I. S., Yeo, G. S., Keogh, J. M., Aminian, S., et al. (2000) Dominant and recessive inheritance of morbid obesity associated with melanocortin 4 receptor deficiency. *J Clin Invest* 106, 271-279
45. Yeo, G. S., Lank, E. J., Farooqi, I. S., Keogh, J., et al. (2003) Mutations in the human melanocortin-4 receptor gene associated with severe familial obesity disrupts receptor function through multiple molecular mechanisms. *Human molecular genetics* 12, 561-574
46. Huang, H., and Tao, Y.-X. (2014) A small molecule agonist THIQ as a novel pharmacoperone for intracellularly retained melanocortin-4 receptor mutants. *International journal of biological sciences* 10, 817
47. Del Giudice, E. M., Cirillo, G., Nigro, V., Santoro, N., et al. (2002) Low frequency of melanocortin-4 receptor (MC4R) mutations in a Mediterranean population with early-onset obesity. *International journal of obesity* 26, 647
48. Reinehr, T., Hinney, A., De Sousa, G., Austrup, F., et al. (2007) Definable somatic disorders in overweight children and adolescents. *The Journal of pediatrics* 150, 618-622. e615
49. Dubern, B., Clément, K., Pelloux, V., Froguel, P., et al. (2001) Mutational analysis of melanocortin-4 receptor, agouti-related protein, and α-melanocyte-stimulating hormone genes in severely obese children. *The Journal of pediatrics* 139, 204-209
50. Cole, S. A., Butte, N. F., Voruganti, V. S., Cai, G., et al. (2010) Evidence that multiple genetic variants of MC4R play a functional role in the regulation of energy expenditure and appetite in Hispanic children. *The American journal of clinical nutrition* 91, 191-199
51. Rene, P., Le Gouill, C., Pogozheva, I. D., Lee, G., et al. (2010) Pharmacological chaperones restore function to MC4R mutants responsible for severe early-onset obesity. *Journal of Pharmacology and Experimental Therapeutics* 335, 520-532
52. Tao, Y.-X., and Huang, H. (2014) Ipsen 5i is a novel potent pharmacoperone for intracellularly retained melanocortin-4 receptor mutants. *Frontiers in endocrinology* 5, 131
53. Vollbach, H., Brandt, S., Lahr, G., Denzer, C., et al. (2017) Prevalence and phenotypic characterization of MC4R variants in a large pediatric cohort. *International Journal of Obesity* 41, 13-22

54. Wangensteen, T., Kolsgaard, M. P., Mattingsdal, M., Joner, G., et al. (2009) Mutations in the melanocortin 4 receptor (MC4R) gene in obese patients in Norway. *Experimental and clinical endocrinology & diabetes* 117, 266-273
55. Wang, Z.-Q., and Tao, Y.-X. (2011) Functional studies on twenty novel naturally occurring melanocortin-4 receptor mutations. *Biochimica et Biophysica Acta (BBA)-Molecular Basis of Disease* 1812, 1190-1199
56. Tan, K., Pogozheva, I. D., Yeo, G. S., Hadaschik, D., et al. (2009) Functional characterization and structural modeling of obesity associated mutations in the melanocortin 4 receptor. *Endocrinology* 150, 114-125
57. Delhanty, P. J., Bouw, E., Huisman, M., Vervenne, R. M., et al. (2014) Functional characterization of a new human melanocortin-4 receptor homozygous mutation (N72K) that is associated with early-onset obesity. *Molecular biology reports* 41, 7967-7972
58. Nowacka-Woszuk, J., Cieslak, J., Skowronska, B., Majewska, K. A., et al. (2011) Missense mutations and polymorphisms of the MC4R gene in Polish obese children and adolescents in relation to the relative body mass index. *Journal of applied genetics* 52, 319-323
59. Pérez Jurado, L. A., Granell, S., Serra Juhé, C., Martos Moreno, G. Á., et al. (2012) A novel melanocortin-4 receptor mutation MC4R-P272L associated with severe obesity has increased propensity to be ubiquitinated in the ER in the face of correct folding. *PLoS ONE. 2012; 7 (12): e50894*
60. Clement, K., Biebermann, H., Farooqi, I. S., Van der Ploeg, L., et al. (2018) MC4R agonism promotes durable weight loss in patients with leptin receptor deficiency. *Nature medicine* 24, 551-555
61. Hinney, A., Hohmann, S., Geller, F., Vogel, C., et al. (2003) Melanocortin-4 receptor gene: case-control study and transmission disequilibrium test confirm that functionally relevant mutations are compatible with a major gene effect for extreme obesity. *The Journal of Clinical Endocrinology & Metabolism* 88, 4258-4267
62. Nijenhuis, W. A., Garner, K. M., van Rozen, R. J., and Adan, R. A. (2003) Poor cell surface expression of human melanocortin-4 receptor mutations associated with obesity. *Journal of Biological Chemistry* 278, 22939-22945
63. Biebermann, H., Krude, H., Elsner, A., Chubanov, V., et al. (2003) Autosomal-dominant mode of inheritance of a melanocortin-4 receptor mutation in a patient with severe early-onset obesity is due to a dominant-negative effect caused by receptor dimerization. *Diabetes* 52, 2984-2988
64. LUBRANO-BERTHELIER, C., Cavazos, M., Dubern, B., Shapiro, A., et al. (2003) Molecular genetics of human obesity-associated MC4R mutations. *Annals of the New York Academy of Sciences* 994, 49-57
65. Srinivasan, S., Santiago, P., Lubrano, C., Vaisse, C., and Conklin, B. R. (2007) Engineering the Melanocortin-4 Receptor to Control Constitutive and Ligand-Mediated Gs Signaling In Vivo. *PLOS ONE* 2, e668
66. Govaerts, C., Srinivasan, S., Shapiro, A., Zhang, S., et al. (2005) Obesity-associated mutations in the melanocortin 4 receptor provide novel insights into its function. *Peptides* 26, 1909-1919
67. Gu, W., Tu, Z., Kleyn, P. W., Kissebah, A., et al. (1999) Identification and functional analysis of novel human melanocortin-4 receptor variants. *Diabetes* 48, 635-639
68. Ho, G., and MacKenzie, R. G. (1999) Functional characterization of mutations in melanocortin-4 receptor associated with human obesity. *J Biol Chem* 274, 35816-35822
69. Thearle, M. S., Muller, Y. L., Hanson, R. L., Mullins, M., et al. (2012) Greater impact of melanocortin-4 receptor deficiency on rates of growth and risk of type 2 diabetes during childhood compared with adulthood in Pima Indians. *Diabetes* 61, 250-257
70. Melchior, C., Schulz, A., Windholz, J., Kiess, W., et al. (2012) Clinical and functional relevance of melanocortin-4 receptor variants in obese German children. *Hormone research in paediatrics* 78, 237-246
71. Rovite, V., Petrovska, R., Vaivade, I., Kalnina, I., et al. (2014) The role of common and rare MC4R variants and FTO polymorphisms in extreme form of obesity. *Molecular biology reports* 41, 1491-1500

72. Granell, S., Serra-Juhe, C., Martos-Moreno, G. A., Díaz, F., et al. (2012) A novel melanocortin-4 receptor mutation MC4R-P272L associated with severe obesity has increased propensity to be ubiquitinated in the ER in the face of correct folding. *PloS one* 7, e50894
73. Alharbi, K. K., Spanakis, E., Tan, K., Smith, M. J., et al. (2007) Prevalence and functionality of paucimorphic and private MC4R mutations in a large, unselected European British population, scanned by meltMADGE. *Human mutation* 28, 294-302
74. Kobayashi, H., Ogawa, Y., Shintani, M., Ebihara, K., et al. (2002) A novel homozygous missense mutation of melanocortin-4 receptor (MC4R) in a Japanese woman with severe obesity. *Diabetes* 51, 243-246
75. Huang, H., and Tao, Y. X. (2012) Pleiotropic functions of the transmembrane domain 6 of human melanocortin-4 receptor. *J Mol Endocrinol* 49, 237-248
76. Buchbinder, S., Bärtsch, U., Müller, M., Zorn, M., et al. (2011) A novel missense mutation T101N in the melanocortin-4 receptor gene associated with obesity. *Genet Mol Res* 7, 1042-1049
77. Jacobson, P., Ukkola, O., Rankinen, T., Snyder, E. E., et al. (2002) Melanocortin 4 receptor sequence variations are seldom a cause of human obesity: the Swedish Obese Subjects, the HERITAGE Family Study, and a Memphis cohort. *The Journal of Clinical Endocrinology & Metabolism* 87, 4442-4446
78. Valli-Jaakola, K., Lipsanen-Nyman, M., Oksanen, L., Hollenberg, A. N., et al. (2004) Identification and characterization of melanocortin-4 receptor gene mutations in morbidly obese Finnish children and adults. *The Journal of Clinical Endocrinology & Metabolism* 89, 940-945
79. Heck, M., Schädel, S. A., Maretzki, D., Bartl, F. J., et al. (2003) Signaling States of Rhodopsin Formation of the storage form, metarhodopsin III, from active metarhodopsin II. *Journal of Biological Chemistry* 278, 3162-3169
80. Buono, P., Pasanisi, F., Nardelli, C., Ieno, L., et al. (2005) Six novel mutations in the proopiomelanocortin and melanocortin receptor 4 genes in severely obese adults living in southern Italy. *Clinical chemistry* 51, 1358-1364
81. Tarnow, P., Rediger, A., Brumm, H., Ambrugger, P., et al. (2008) A heterozygous mutation in the third transmembrane domain causes a dominant-negative effect on signalling capability of the MC4R. *Obes Facts* 1, 155-162
82. Deliard, S., Panossian, S., Mentch, F. D., Kim, C. E., et al. (2013) The missense variation landscape of FTO, MC4R, and TMEM18 in obese children of African Ancestry. *Obesity* 21, 159-163
83. Rouskas, K., Meyre, D., Stutzmann, F., Paletas, K., et al. (2012) Loss-of-Function Mutations in MC4R Are Very Rare in the Greek Severely Obese Adult Population. *Obesity* 20, 2278-2282
84. Rettenbacher, E., Tarnow, P., Brumm, H., Prayer, D., et al. (2007) A novel non-synonymous mutation in the melanocortin-4 receptor gene (MC4R) in a 2-year-old Austrian girl with extreme obesity. *Experimental and clinical endocrinology & diabetes* 115, 7-12
85. Dubern, B., Bisbis, S., Talbaoui, H., Le Beyec, J., et al. (2007) Homozygous null mutation of the melanocortin-4 receptor and severe early-onset obesity. *The Journal of pediatrics* 150, 613-617. e611
86. Censani, M., Conroy, R., Deng, L., Oberfield, S. E., et al. (2014) Weight loss after bariatric surgery in morbidly obese adolescents with MC4R mutations. *Obesity* 22, 225-231
87. Lee, Y. S., Poh, L. K. S., Kek, B. L. K., and Loke, K. Y. (2008) Novel melanocortin 4 receptor gene mutations in severely obese children. *Clinical endocrinology* 68, 529-535
88. Saeed, S., Butt, T. A., Anwer, M., Arslan, M., and Froguel, P. (2012) High prevalence of leptin and melanocortin-4 receptor gene mutations in children with severe obesity from Pakistani consanguineous families. *Molecular genetics and metabolism* 106, 121-126
89. Ochoa, M. C., Azcona, C., Biebermann, H., Brumm, H., et al. (2007) A novel mutation Thr162Arg of the melanocortin 4 receptor gene in a Spanish children and adolescent population. *Clinical endocrinology* 66, 652-658
90. Jelin, E., Daggag, H., Speer, A., Hameed, N., et al. (2016) Melanocortin-4 receptor signaling is not required for short-term weight loss after sleeve gastrectomy in pediatric patients. *International Journal of Obesity* 40, 550

91. Ma, L., Tataranni, P. A., Bogardus, C., and Baier, L. J. (2004) Melanocortin 4 receptor gene variation is associated with severe obesity in Pima Indians. *Diabetes* 53, 2696-2699
92. Ma, L., Tataranni, P. A., Bogardus, C., and Baier, L. J. (2004) Melanocortin 4 receptor gene variation is associated with severe obesity in Pima Indians. *Diabetes* 53, 2696-2699
93. Krakoff, J., Ma, L., Kobes, S., Knowler, W. C., et al. (2008) Lower metabolic rate in individuals heterozygous for either a frameshift or a functional missense MC4R variant. *Diabetes* 57, 3267-3272
94. Alfieri, A., Pasanisi, F., Salzano, S., Esposito, L., et al. (2010) Functional analysis of melanocortin-4-receptor mutants identified in severely obese subjects living in Southern Italy. *Gene* 457, 35-41
95. Song, J., Wang, D., Ma, J., and Wang, H. (2015) Mutation screening and function prediction of melanocortin-4 receptor gene in obese children. *Zhongguo dang dai er ke za zhi= Chinese journal of contemporary pediatrics* 17, 356-361
96. Beckers, S., Zegers, D., de Freitas, F., Peeters, A. V., et al. (2010) Identification and functional characterization of novel mutations in the melanocortin-4 receptor. *Obesity facts* 3, 304-311
97. Kim, D.-H., Shin, S. W., and Baik, J.-H. (2008) Role of third intracellular loop of the melanocortin 4 receptor in the regulation of constitutive activity. *Biochemical and biophysical research communications* 365, 439-445
98. Aslan, I. R., Campos, G. M., Calton, M. A., Evans, D. S., et al. (2011) Weight loss after Roux-en-Y gastric bypass in obese patients heterozygous for MC4R mutations. *Obesity surgery* 21, 930-934
99. Reinehr, T., Hebebrand, J., Friedel, S., Toschke, A. M., et al. (2009) Lifestyle intervention in obese children with variations in the melanocortin 4 receptor gene. *Obesity* 17, 382-389
100. Lubrano-Berthelier, C., Cavazos, M., Le Stunff, C., Haas, K., et al. (2003) The human MC4R promoter: characterization and role in obesity. *Diabetes* 52, 2996-3000
101. Shao, X., Jia, W., Cai, S., Fang, Q., et al. (2005) Cloning and functional analysis of melanocortin 4 receptor mutation gene F261S. *Zhonghua yi xue za zhi* 85, 366-369
102. Farooqi, I. S., Yeo, G. S., Keogh, J. M., Aminian, S., et al. (2000) Dominant and recessive inheritance of morbid obesity associated with melanocortin 4 receptor deficiency. *The Journal of clinical investigation* 106, 271-279
103. Mergen, M., Mergen, H., Ozata, M., Oner, R., and Oner, C. (2001) Rapid communication: a novel melanocortin 4 receptor (MC4R) gene mutation associated with morbid obesity. *The Journal of Clinical Endocrinology & Metabolism* 86, 3448-3448
104. He, S., and Tao, Y.-X. (2014) Defect in MAPK signaling as a cause for monogenic obesity caused by inactivating mutations in the melanocortin-4 receptor gene. *International journal of biological sciences* 10, 1128
105. Doulla, M., McIntyre, A. D., Hegele, R. A., and Gallego, P. H. (2014) A novel MC4R mutation associated with childhood-onset obesity: A case report. *Paediatrics & child health* 19, 515-518
106. Roth, C. L., Ludwig, M., Woelfle, J., Fan, Z.-C., et al. (2009) A novel melanocortin-4 receptor gene mutation in a female patient with severe childhood obesity. *Endocrine* 36, 52-59
107. Tao, Y. X. (2009) Mutations in Melanocortin-4 Receptor and Human Obesity. *Progress in molecular biology and translational science* 88, 173-204
108. Santini, F., Maffei, M., Ceccarini, G., Pelosini, C., et al. (2004) Genetic screening for melanocortin-4 receptor mutations in a cohort of Italian obese patients: description and functional characterization of a novel mutation. *The Journal of Clinical Endocrinology & Metabolism* 89, 904-908
109. VanLeeuwen, D., Steffey, M. E., Donahue, C., Ho, G., and MacKenzie, R. G. (2003) Cell surface expression of the melanocortin-4 receptor is dependent on a C-terminal di-isoleucine sequence at codons 316/317. *Journal of Biological Chemistry* 278, 15935-15940
110. Fan, Z. C., Sartin, J. L., and Tao, Y. X. (2008) Molecular cloning and pharmacological characterization of porcine melanocortin-3 receptor. *J Endocrinol* 196, 139-148
111. Ballesteros, J. A., and Weinstein, H. (1995) Integrated Methods for the Construction of Three-Dimensional Models and Computational Probing of Structure-Function Relationships in G-Protein Coupled Receptors. *Methods Neurosci* 25, 366-428

112. Ballesteros, J. A., Weinstein, H., and Stuart, C. S. (1995) [19] Integrated methods for the construction of three-dimensional models and computational probing of structure-function relations in G protein-coupled receptors. in *Methods in Neurosciences*, Academic Press. pp 366-428
113. Ersoy, B. A., Pardo, L., Zhang, S., Thompson, D. A., et al. (2012) Mechanism of N-terminal modulation of activity at the melanocortin-4 receptor GPCR. *Nat Chem Biol* 8, 725-730
114. Lensing, C. J., Adank, D. N., Wilber, S. L., Freeman, K. T., et al. (2017) A Direct in Vivo Comparison of the Melanocortin Monovalent Agonist Ac-His-DPhe-Arg-Trp-NH2 versus the Bivalent Agonist Ac-His-DPhe-Arg-Trp-PEDG20-His-DPhe-Arg-Trp-NH2: A Bivalent Advantage. *ACS chemical neuroscience* 8, 1262-1278
115. Lensing, C. J., Freeman, K. T., Schnell, S. M., Adank, D. N., et al. (2016) An in Vitro and in Vivo Investigation of Bivalent Ligands That Display Preferential Binding and Functional Activity for Different Melanocortin Receptor Homodimers. *J Med Chem* 59, 3112-3128
